# External supply risk of agricultural products trade along the Belt and Road under the background of COVID-19

**DOI:** 10.3389/fpubh.2023.1122081

**Published:** 2023-02-15

**Authors:** Qiuguang Hu, Mengqian Guo, Fang Wang, Liqun Shao, Xinyi Wei

**Affiliations:** ^1^College of Business, Ningbo University, Ningbo, Zhejiang, China; ^2^Dong Hai Strategic Research Institute, Ningbo University, Ningbo, Zhejiang, China; ^3^College of Economics, Zhejiang University of Technology, Hangzhou, Zhejiang, China; ^4^Business School, Leeds Bechett University, Leeds, United Kingdom; ^5^College of Economics and Management, Northwest A&F University, Yangling, Shanxi, China

**Keywords:** COVID-19 pandemic, agricultural products trade, external supply risk, complex network analysis, Belt and Road

## Abstract

Agricultural product trade along the Belt and Road (B&R) is an important part of the international food security system, the vulnerabilities of which have been highlighted by the recent COVID-19 pandemic. Based on the complex network analysis, this study analyzes the characteristics of agricultural products trade network along the B&R. It also combines the effects of COVID-19 with the import trade volume of agricultural products in countries along the B&R to build a risk supply model of agricultural products. The results show that: (1) In 2021, the spatial correlation structure of agricultural products trade along the B&R became increasingly sparse, and the network connectivity and density also decreased. (2) The network showed obvious scale-free distribution characteristics and obvious heterogeneity. Five communities emerged under the influence of the core node countries, but the formation of community in 2021 had obvious geopolitical characteristics. (3) Under the influence of the COVID-19 epidemic, the number of countries with medium-risk and high-risk level along the route facing external dependence risk (R_EDI_), import concentration risk (R_HHI_) and COVID-19 epidemic risk (R_RICI_) increased in 2021, and the number of countries with extremely low-risk level decreased. (4) The dominant risk type of external supply of agricultural products along the route changed from compound risk type in 2019 to epidemic risk in 2021. Hence, the results can be expected to prevent external risk impact from reducing excessive concentration of agricultural products trade and excessive dependence on the external market.

## 1. Introduction

Given the mismatch of agricultural product supply and COVID-19 pandemic, the trade of agricultural products has become an important link in stabilizing global agricultural products trade network and ensuring food security (1). The B&R agricultural products trade network covering 140 countries is an essential part of the entire global trade network and provides a guarantee for the safety of global agricultural products and sustainable utilization of its resources. International trade in the agriculture industry, on the one hand, guarantees global food safety. On the other hand, it also makes the global food system increasingly complicated and may even increase a country's susceptibility to external interference (2).

This increased vulnerability was seen in how the recent outbreak of the COVID-19 pandemic exposed the fragility of agricultural product supply along the B&R and subsequently triggered a food crisis. Afterwards, more than 20 countries in the world successively introduced management measures to restrict or upgrade grain exports, most of which are concentrated in the B&R and are comprised of China's largest agricultural products trading partners such as Russia, Ukraine, and Kazakhstan. National policies, food sovereignty and global trade are playing an increasingly important role in ensuring food security (3). These countries' restrictions on grain exports certainly have an impact on the agricultural products trade network and food security along the route, and then transmit and influence the global food security. According to The State of World Food Security and Nutrition in 2021 the beginning of the COVID-19 pandemic saw approximately 720 to 811 million people worldwide suffer from hunger in 2020. Addressing food security challenges requires the participation of different sector actors and joint efforts to sustainably improve food insecurity. At a time of dramatic global and local change, policy makers and decision makers face difficult choices in improving food security (4).

While COVID-19 continues to spread and affect global food supply and industrial chains, it has also exposed numerous glaring drawbacks in global food security governance. After the decade long development of the Belt and Road Initiative (BRI), countries along the route have become highly co-dependent with the trade of agricultural products in likewise becoming increasingly complicated, thus resulting in the external supply risk of agricultural products being inevitably affected by both global market forces and COVID-19. According to the strategic demand of avoiding external supply risks of agricultural products along the B&R and ensuring food security, this study illustrated the status and interdependence of different countries in the B&R's agricultural products trade network from a complex trade network perspective. It also identified the source of its external supply risk to provide scientific decision for the sustainable supply of agricultural products in countries along the B&R.

Analyzing the structure and evolution mode of trade network helps understand the sensitivity of agricultural product trade in countries along the B&R. Simultaneously, it is necessary to deconstruct how the said trade network connects the countries along the route through the flow of its products. Complex network theory constructs a network based on nodes and connections and reveals its small-world characteristics and scale-free distribution properties (5). With the theory's development, scholars have forwarded novel concepts and metrics such as degree distribution, condensed subgroups, and network correlation (6, 7). All being increasingly used to study the overall trade network of B&R (8–11) including commodities such as energy (12, 13), mechanical and electrical products (14), and especially, agricultural products (15–17), among others.

Given the shifts in the global status quo, the issue of food security has attracted increasing attention from scholars. Some studies have begun using complex network analysis to build food trade networks using grain or single grain variety as the research object to explore the structural characteristics and dynamic changes of the network (18–20). The abovementioned research on both regional trade and specific products shows that the connection of network structure is getting increasingly closer, hence the influence and control of core countries in the network have been gradually increasing as well. Community structures with stronger internal cooperation and competition and are centered on core node countries promotes the global trade network to develop into a “strong but fragile” form (21).

When major exporting countries have shortages or restrict their exports in the agricultural products trading market, the trade network likewise becomes more vulnerable. Moreover, with the advancement of regional integration, node countries in the same module tend to have closer trade ties and stronger dependence. However, this does not automatically mean that the node countries in the same modularity have more trade advantages, node members in the same module even tend to be more vulnerable to risks due to geographical proximity. Nonetheless, extant studies mostly explore the close connection between nodes in the trade network as the structural advantage, therein ignoring the negative impact of uncertain risks on this closely linked trade structure.

With the outbreak of the COVID-19 pandemic in 2020, most countries restricted agricultural product trade for domestic security, which impacted the stability of agricultural products trade network along the B&R and deepened concerns on agricultural products import concentration. Generally, when a country imports agricultural products from more countries, the risk of agricultural products trade can be, to some extent, dispersed, but the risk of external supply has perpetually been a scholarly focus for those in the field of resource supply (22–24). To study external supply risk, scholars forward the external supply risk index model which combines the Herfindahl-Hirschman index, import and external dependence index, and other indices. The model has now been widely used in analyzing food, energy, and other related issues in various disciplines (25, 26).

Nevertheless, the COVID-19 pandemic further exposed already existing vulnerabilities of external supply, especially in import concentration and the over-reliance on external supply. Currently, most studies are still based on the Herfindahl-Hirschman and Shannon-Wiener indices to study external supply risk, with the COVID-19 epidemic index rarely being included in the external supply risk model, even from the perspective of the pandemic itself. Data used in these extant studies also do not cover trade data after the outbreak of COVID-19 (27), making it difficult to explore the pandemic's effects on trade networks as an external supply risk.

Given these theoretical gaps, this study follows the perspective of complex network and focuses on exploring the external supply risks of agricultural products in the B&R. It uses the import trade data of agricultural products in 2019 and 2021 from countries along the B&R to study the following: (1) Using complex network analysis indicators, this study depicts the characteristics of trade network, compares the development of trade networks of countries along B&R before and after the outbreak of COVID-19 epidemic, and identifies the core node countries in the network. It then analyzes the flow pattern of agricultural products along the route and the dependence of node countries by modularity; (2) Considering the effects of the COVID-19 pandemic as an external risk to agricultural product trade, the current study constructs an external agricultural product supply risk model comprised of the Herfindahl-Hirschman, import dependence, and COVID-19 risk indices of importing countries to evaluate the internal and national level shifts of external supply risks in the B&R. It also analyzes the leading factors of these external supply risks. Clarifying these problems contributes to find out the influence of COVID-19 on agricultural products trade network between China and the B&R along with the degree of influence on food supply security in the B&R. This ultimately provides targeted policy for countries along the B&R to deal with the risk of COVID-19 on external food supply.

Compared to previous evaluation of external supply risk of trade which considers risk factors such as political stability and economic stability, this paper pioneeringly sets the research background under the COVID-19 pandemic and juxtaposes the trade along the B&R which is facing extremely strict control and interruption in the import and export of goods and services. Therefore, it is more realistic for the research to incorporate the risk of infectious diseases into the external supply risk evaluation model.

The risk assessment model constructed herein is also conveniently assesses the impact of COVID-19 on the import and export of agricultural products by incorporating the RICI into the AECSI index. By calculating and comparing external risk assessment models before and after COVID-19, it is crystal clear that the external supply risks of agricultural products trade in countries along the B&R are easily affected by RICI risks, and the control measures accompanying the COVID-19 outbreak have strengthened the external supply risks of agricultural products trade. Therefore, this demonstrates the importance of avoiding inappropriate control measures when engaging in trade.

## 2. Research methods and data sources

### 2.1. Research methods

#### 2.1.1. Complex network analysis method

##### 2.1.1.1. Complex network construction

The analysis of the overall characteristics of complex network and its correlative indicators are all binary matrices based on the transformation of original data. Following Gleditsch (28) a directional network model was built with countries as network node. The network is calculated as follows:


$$\begin{array}{l} \;G=\left(IM,\;A\right),\\ IM_{i,j}=[IM_{i,j}^{t}](i=1,2......88;j=1,2.....88;t=2019,2021,)\\ \;A=[a_{i,j}](i=1,2.......88;j=1,2......88) \end{array}$$

where *G* = (*IM, A*) represents the agricultural products trade network of countries along the B&R, *IM*_*i,j*_ represents the total agricultural products trade volume between *i* and *j*, the adjacency matrix *A* represents the trade relationship between *i* and *j*, the binary adjacency matrix represents the relationship between the node countries in the network. The transformation of the binary matrix must set an appropriate threshold, otherwise, there will be a deviation in the result of binary transformation due to the huge gap in the total trade volume of agricultural products among B&R countries, this paper takes the total import trade of agricultural products in countries along the B&R as the basic data, and takes 100 million USD as the standard (29).

##### 2.1.1.2. Degree

Degree refers to the number of countries directly connected with a node country in the agricultural product trade network, which is defined as $DC\left(x\right)=\underset{j}{\sum }x_{i,j}\left(x\right)$ and includes both the out-degree and in-degree in the directed network. Degree is mainly used to analyze the degree to which the node countries along the route are in the central position in the agricultural products trade network: the higher the degree, the more the node countries have contact with other countries in the agricultural products trade network of the B&R. The more central the node countries are in the network, the greater influence they have on the trade network (30).

##### 2.1.1.3. Modularity analysis

Modularity analysis in the complex network refers to dividing all the nodes in a trade network into several independent (but internally connected) modules and judging the information of the whole network structure by analyzing the relationships among the “blocks” (31). The calculation formula of modularity degree is $Q=\frac{1}{2m}\underset{i,j}{\sum }\left[A_{ij}-\frac{kikj}{2m}\right]\delta \left(c_{i},c_{j}\right)$, *A*_*ij*_ represents the weight of edges between node *i* and node *j*. When the network is not weighted, the weight of all edges can be regarded as 1.$k_{i}=\underset{j}{\sum }A_{ij}$ indicates the sum of weights of all edges connected to node *i*; δ(*c*_*i*_, *c*_*j*_) indicates that node *i* and node *j* belong to the same community; $m=\frac{1}{2}\underset{ij}{\sum }A_{ij}$ represents the sum of the weights of all edges. Modularity analysis is mainly based on the number of connections between source and target to divide the modules. Afterwards, it analyzes the flow of major agricultural products trade relations in the modules and the change in interdependence within countries in the modules.

#### 2.1.2. The external supply risk model

The construction of external risk supply model is based on three indices: the external dependence index, the Herfindahl-Hirschman index, and the risk of COVID-19 index from importing countries (2).

(1) Agricultural product import external dependence (EDI) is the ratio of agricultural product import and gross domestic product (GDP), expressed as $EDI_{i}=\frac{QI_{ij}}{GDP_{ij}}$. *QI*_*ij*_ indicates the agricultural product import volume of country *i* in year *j*, and *GDP*_*ij*_ indicates the GDP of country *i* in year *j*. In order to overcome the influence of the negative values, a value of 1 was assigned if *EDI*_*ij*_ < 0, If *EDI*_*ij*_>0, then a a value of *EDI*_*ij*_+1 was assigned. External dependence risk is herein defined as R _EDI_.
(2) The Herfindahl-Hirschman Index (HHI), also known as the import concentration index, describes the import concentration (32), is defined as $HHI_{i}=\sum_{i}(\frac{IQ_{ijk}}{IQ_{ij}}$)^2^ (33), and *IQ*_*ijk*_ indicates the total amount of agricultural products imported by country *i* from *k* in year *j* and the *IQ*_*ij*_ represents total amount of imports of country *i* in year *j*. Import concentration risk was represented by the variable *R*_*HHI*_.
(3) The COVID-19 risk index (RICI) is defined as $RICI=\underset{i}{\sum }{\left(\frac{IQ_{ijk}}{IQ_{ij}}\right)}^{*}CRI_{i}$. *CRI*_*i*_ is COVID-19 risk index of country *i*. Here, the COVID-19 risk index of the importing country is represented as *R*_*RICI*_. There were no COVID-19 outbreaks in 2019, hence the COVID-19 risk index was assigned as 1. Following the GIS method of natural discontinuity, *R*_*EDI*_*, R*_*HHI*_ and *R*_*RICI*_ are divided into five grades.

Given that the ranges of the above evaluation indexes are close and the difference in the degree of impact on agricultural products supply is small, this study further establishes a consistent agricultural product supply risk index. Here, the abovementioned indices are divided by equal interval method, and then the standard risk factor values are obtained. *AECSI*_*i*_ = *R*_*EDI*_ + *R*_*HHI*_ + *R*_*RICI*_ was calculated using the summation method. According to the range of AECSI results, the external agricultural product supply risks are divided into five grades by equal interval method (Table 1). Following the proportion of *R*_*EDI*_*, R*_*HHI*_ and *R*_*RICI*_ scores in *R*_*ECSI*_, calculate the contribution rate of each evaluation factor to *R*_*ECSI*_ was calculated and recorded as CREDI, CR_HHI_, and CR_RICI_ respectively. Finally, the type of external supply risk of agricultural products was determined (Table 2).

**Table 1:** Natural breaks of different risk levels in 2019.

**Level**	**Natural breaks**			
	**R**_EDI_	**R**_HHI_	**R**_RICI_	**AECSI**
Very low	1.0051–1.0253	1.0140–1.15580	0–0.6248	0–0.5000
low	1.0280–1.0453	1.1558–1.4158	0.6248–1.7383	0.5000–4.3216
Medium	1.0481–1.0670	1.4158–1.8543	1.7383–3.0959	4.3216–5.2481
High	1.0697–1.1010	1.8543–2.3412	3.0959–5.2514	5.2481–7.3183
Very high	1.1113–1.1496	2.3412–3.5616	5.2514–8.4550	7.3183–10.5108

**Table 2:** Criteria for classification into dominant risk factors.

**Classification**	**Type**
CR_DEI_ > 50%	EDI risk
CR_HHI_ > 50%	HHI risk
CR_RICI_ > 50%	RICI risk
CR_EDI_, CR_HHI_和CR_RICI_ < 50%	Compound risks

### 2.2. Data

#### 2.2.1. Trade network data

The agricultural products studied herein are mainly based on the definition laid out by the WTO, with products from chapters 01–24 and 41, 43, 50, 51 and 52 under HS two-digit code selected as the main research objects. Trade data was extracted from the UN comtrade database (Download trade data | UN Comtrade: International Trade Statistics). The import volume of agricultural products was also used to build a trade network. The scope of the B&R was defined as the 140 countries^1^ along the B&R following the GREEN BELT AND ROAD INITIATIVE CENTER [“一带一路”沿线国家 - Green Belt and Road Initiative Center (green-bri.org)]. Eighty eight countries^2^ were chosen as the sample data set based on the availability and completeness of the data, and the network features and visualization were examined using the Ucinet6, Gephi, and ArcGIS software programs.

#### 2.2.2. COVID-19 risk index

The COVID-19 risk data COVID-19 risk index (http://covid19-risk-index.com/) was extracted from the COVID-19 Risk Index Global Epidemic Comprehensive Risk Index jointly published by Trade Wind Technology Jian Chen Team, Huashan Hospital Zhang Wenhong Team, and the Angu Medical Technology team.

## 3. Result

### 3.1. Network characteristics

#### 3.1.1. Network association structure

**Figure 1:**
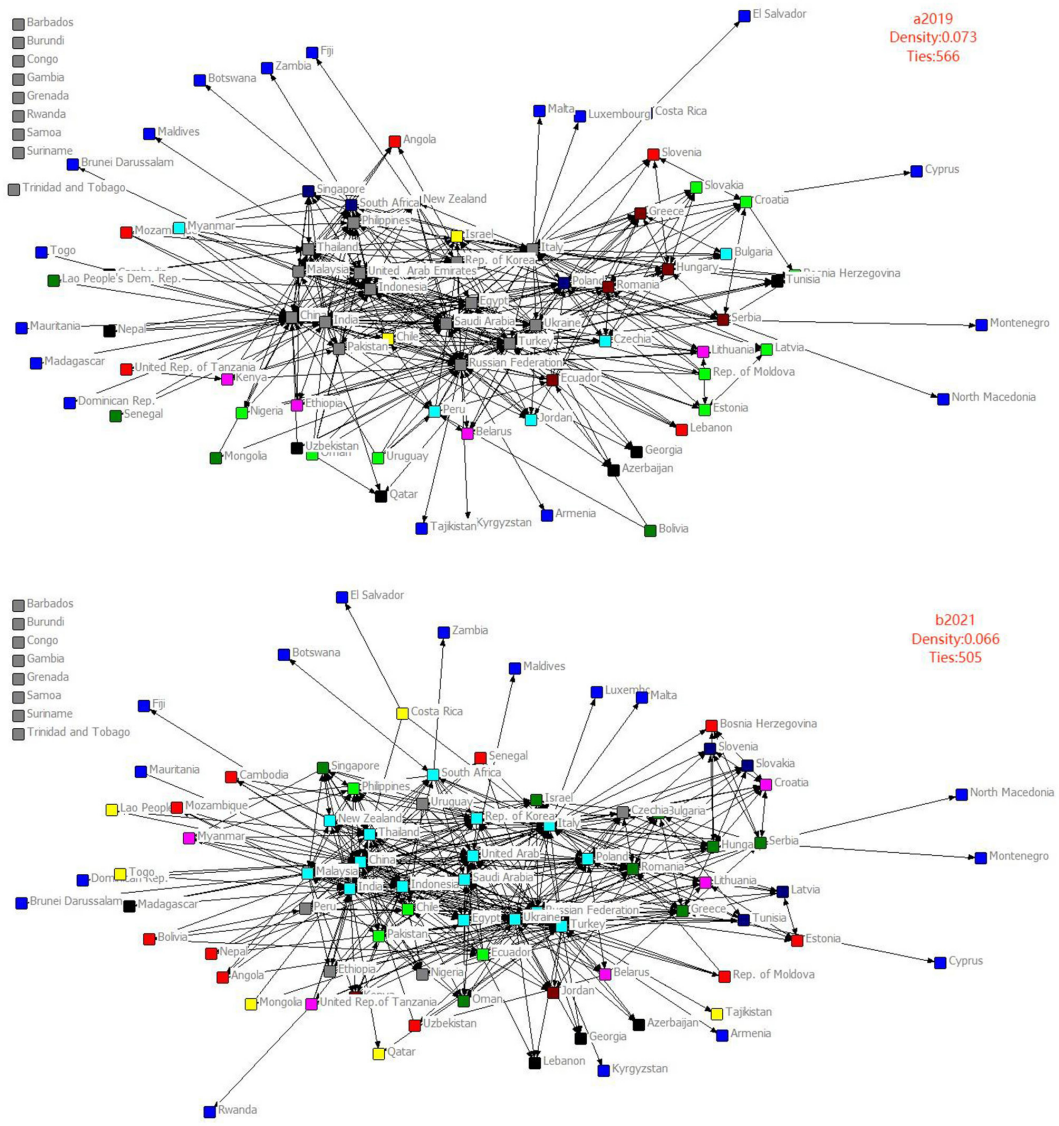
Evolution diagram of agricultural products trade network pattern of countries along the B&R in 2019 & 2021.

To intuitively reflect the changes of agricultural products trade status of countries along the B&R as affected by the COVID-19 pandemic, the directional network topology maps of agricultural products trade of countries along the B&R in 2019 and 2021 were drawn (Figure 1 a2019 and b2021), and the network density and the number of connections were calculated to reflect the evolutionary trend of agricultural products trade network. By comparing a2019 and b2021 in Figure 1, the agricultural trade network of countries along the B&R clearly had the following characteristics:

In the characteristics of the overall network structure, the core-periphery structure of agricultural products trade network along the route had obviously changed. In 2019, the core module included 15 node countries (dark gray points in a2019) such as Russia, Ukraine, United Arab Emirates, and Philippines. By 2021, the number of node countries in the core module increased to 17. However, the number of countries outside the core circle, especially in the marginal zone, significantly increased and the network outside the circle tended to be loose. This may be because countries outside the core circle have relatively weak economic strength and their ability to resist external risks is also relatively weak.

With the sudden onset of the pandemic and the economic blockades of various countries which subsequently followed, it is difficult for countries outside this circle to use the BRI to ensure the import of agricultural products, thereby making them more marginalized in the agricultural products trade network and subsequently creating a more serious food security problem. In contrast, countries in the core circle, even before or after the pandemic, can achieve reliable food import or supply due to their sustainable food security strategy and economic strength. Thus, the impact of COVID-19 on node countries in the core circle is relatively weak. It is worth noting that even though the countries in the core circle are relatively less affected by the epidemic in terms of grain import and export at this stage, the complex international situation and conflicts other than the epidemic still require much notable attention.

Comparing the number of connections between node countries in the network, it can be conceived that after breaking out the COVID-19 epidemic in 2020, there were fewer connections between node countries (556 in 2019 compared to 505 in 2021), indicating a decline in the number of node countries in the network where agricultural products trade with other nations exceeds $100 million. Although the number of countries outside the agricultural products trade network has decreased (example being Rwanda which began moving to the inner circle), the movement toward the inner circle did not change the result that the number of connections between nodes in the overall network decreased. This decrease may be effects of the COVID-19 pandemic, which caused economies along the route to economically shut down to varying degrees. A report by the World Bank in January 2020 before the outbreak of the epidemic saw global poverty rates decreasing every year, which jarring compared to more than 700 million people worldwide facing extreme poverty due to the pandemic. The pandemic marks the first time that global poverty rates increased in the past 20 years and is likely to continue for a long time, which will affect demand for agricultural products.

Meanwhile, the world has entered into a new political situation characterized by “Security priority and Domestic priority.” The pandemic has ushered in a trend of regionalization, fragmentation, and fuller localization and propelled them into new stages, leading countries along the route to restrict the export of agricultural products and prioritizing domestic security. In addition, with the increasingly complicated international situation, the United States has intensified its containment and suppression of China, coupled by the various regional wars which have further aggravated already existing geopolitical cleavages, thus severely curbed the positive effect of the BRI and cascading to the larger agricultural products trade. Furthermore, the strict blockade and control measures adopted by the outbreak of COVID-19 have restricted the transportation of agricultural products *en masse*, further leading to supply side restrictions of agricultural products.

From the density of the overall trade network along the route, the network density dropped from 0.073 in 2019 to 0.066 in 2021, indicating that the agricultural products trade links of countries along the B&R have become increasingly sparse and the positive effects brought by the BRI are being offset by the negative effects caused by the pandemic. Simultaneously, non-B&R countries spearheaded by the United States began conducting various measures to suppress the BRI, which are then coupled by the larger anti-globalization trend and the sprouting of local wars and conflicts, ultimately leading to what was originally an open cooperation platform provided by the BRI becoming largely handicapped to play its intended role.

#### 3.1.2. Degree distribution

**Figure 2:**
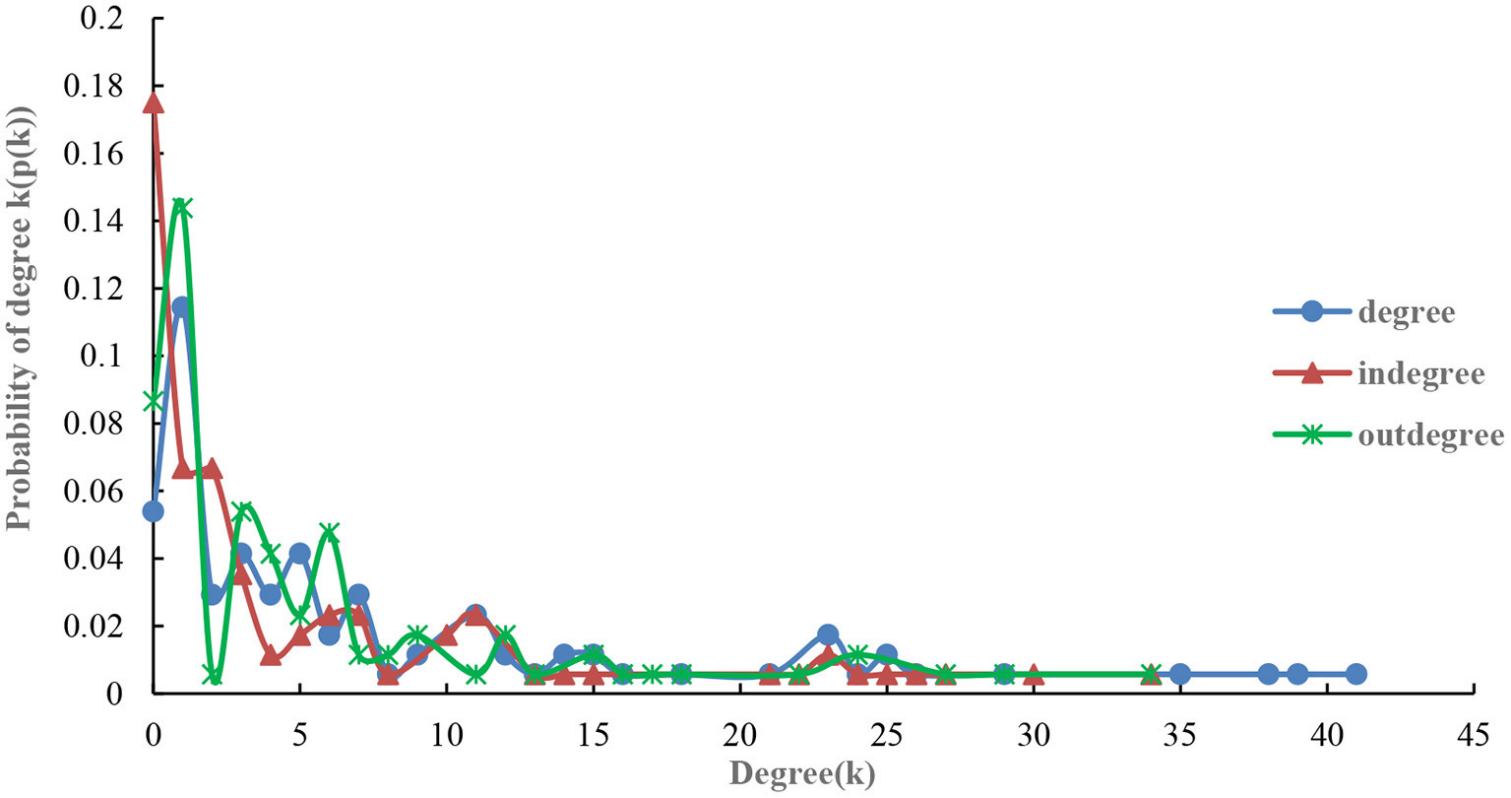
Exponential probability distribution for the degree, in-degree, and out-degree of the import volumes of agricultural products in 2019.

**Figure 3:**
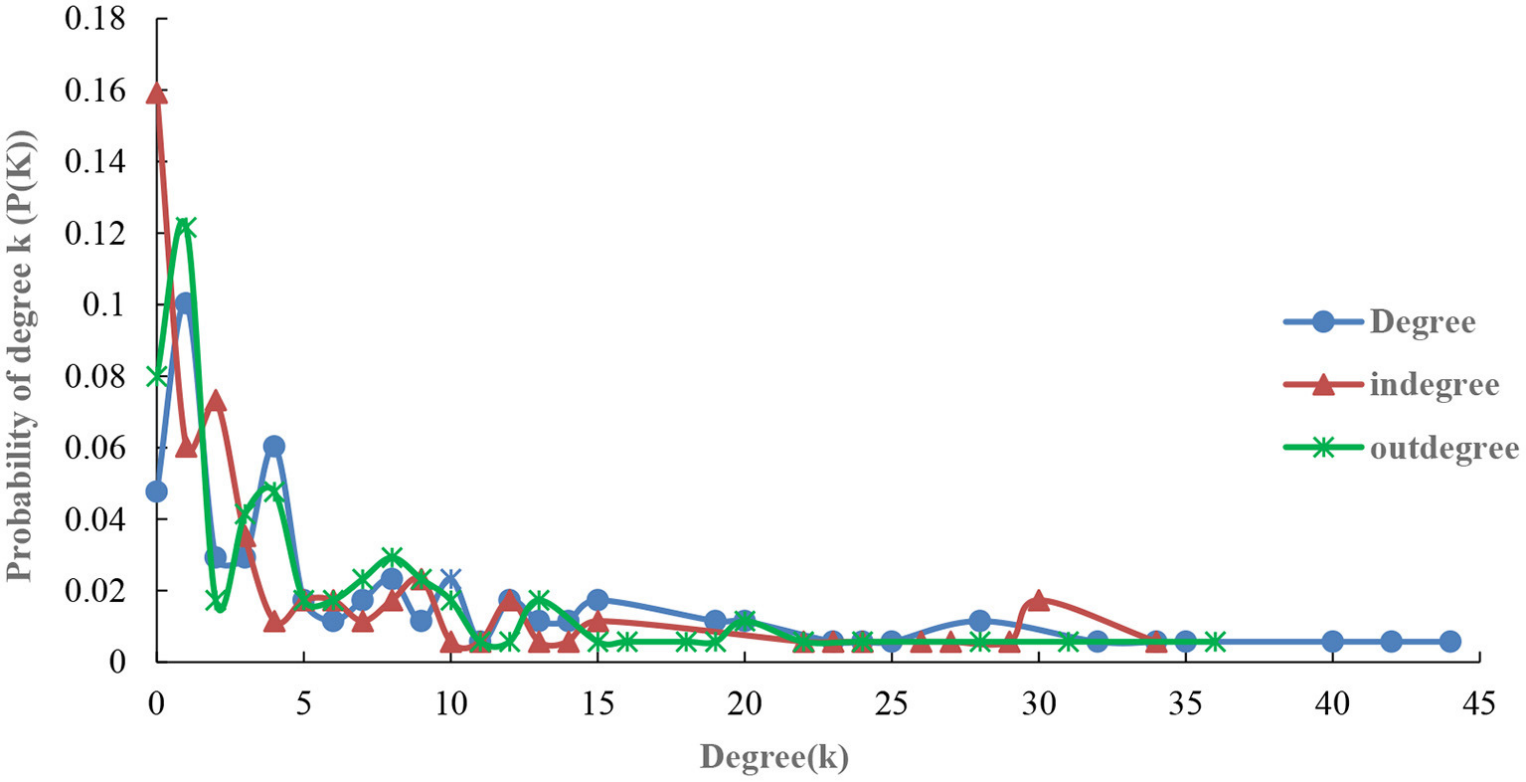
Exponential probability distribution for the degree, in-degree, and out-degree of the import volumes of agricultural product in 2021.

Following the formula above, this study calculates the fitting results of the degree, out-degree, and in-degree along with the exponential probability distribution of countries along the B&R in 2019 and 2021 (see Figures 2, 3). Comparing the results from 2019 and 2021, a few key findings are noted.

From the probability distribution diagram of degree (Figure 2), the exponential probability distribution of degree, in-degree and out-degree of agricultural products trade network along the route in 2019 and 2021 shows a decreasing trend: the number of nodes with higher degree is rare, unlike the number of nodes with lower degree is large, which is an important feature of scale-free distribution network (34). The degree distribution among countries is very uneven with a few nodes with high degree being called “hubs” in scale-free networks, which play a leading role in agricultural products trade networks along the route (35). In 2019 and 2021, the distribution probability of points in the range of 0–15 was the highest and reached more than 80%. This indicated that most node countries in the network are at the relative edge of the agricultural trade network and their ability to control the agricultural trade network is weak. Nonetheless, the distribution probability in 2021 has a longer tail of 40–45, which indicates that there are more node countries with more than 40 agricultural products import trade links in 2021. These findings coincide with the result of network association structure seen in Figure 1.

According to the comparative analysis of both out-degree and in-degree shown in Figure 3, the slope of exponential probability distribution curve of both is steeper in 2019. The probability distribution in the degree distribution range of 0–10 is higher and the probability distribution in the degree distribution range of 20–30 in 2021 is higher. Possible reasons include the following: (1) The COVID-19 outbreak; (2) Agricultural product trade along the B&R being hinged on political factors such as the BRI, APEC, and other organizations; (3) the declining trend of relying on geographical advantages for trade; (4) geographical proximity no longer being the main consideration for agricultural product trade. However, long-distance commodity trade makes the trade market more dispersed (to some extent), hence the number of connections between nodes correspondingly decreases, the probability distribution is concentrated in 0–10, and the degree centrality is relatively low.

Due to the COVID-19 pandemic, some countries began to shift their trade markets to countries with relatively loose pandemic control measures or those who relatively closer in geographical proximity. This made import and export markets become relatively concentrated, with distribution frequency of degrees in the network structure concentrated at 20–30. Following the trade flow of agricultural products along the route (Table 3), the top 10 countries with exports along the route accounted for 59.25% of the total agricultural products exports in 2019, subsequently increasing to 60.49% in 2021. In 2019, the top 10 countries along the route accounted for 53.26% of the total imports of agricultural products along the B&R, which then likewise increased to 56.25% in 2021. Generally, the in-degree network is more heterogeneous than its out-degree counterpart, with core nodes playing a more important role in the network.

**Table 3:** Characteristic of the Agricultural product exporting and importing nations worldwide in 2019 & 2021.

**Rank**	**Export**				**Import**			
	**Country**	**Quantity (Dollars)**	**Proportion (%)**	**Out-degree**	**Country**	**Quantity (Dollars)**	**Proportion (%)**	**In-degree**
**2019**								
1	CHN	28388004154	9.24%	34	CHN	56889520197	18.5%	30
2	IDN	23753536126	7.73%	13	RUS	18972349720	6.12%	27
3	IND	20098378587	6.54%	16	ITA	17691707314	5.76%	34
4	THA	18712887461	6.09%	15	KOR	12460848062	4.95%	8
5	RUS	17104700703	5.56%	27	EGY	12245815698	3.98%	10
6	UKR	15917418319	5.18%	7	IND	12032144998	3.81%	26
7	ITA	15751194738	5.12%	29	TUR	11703304683	3.81%	23
8	MYS	14731175035	4.79%	12	SAU	11527055895	3.75%	4
9	NZL	14702055772	4.78%	6	IDN	9985954133	3.25%	24
10	POL	12968594457	4.22%	15	ARE	9655109871	3.14%	7
/	TOTAL	307384908993	59.25%	/	TOTAL	307384908993	53.26%	/
**2021**								
1	IDN	35888649919	9.41%	13	CHN	78552306715	20.6%	29
2	CHN	28794103903	7.55%	36	RUS	21190263218	5.56%	30
3	IND	26770803485	7.02%	20	ITA	19618077459	5.14%	34
4	THA	23689595579	6.21%	15	IND	18058426891	4.73%	30
5	RUS	22983726395	6.02%	28	TUR	15280510267	4%	26
6	MYS	21401527961	5.61	13	KOR	14746958645	3.86%	9
7	UKR	20716309523	5.43%	10	SAU	13412083539	3.51%	3
8	ITA	17911740180	4.69%	31	MYS	12039061430	3.16%	23
9	NZL	17307137156	4.54%	7	IDN	11266313642	2.95%	27
10	POL	15300714747	4.01%	20	ARE	10454229247	2.74%	8
/	TOTAL	381582788232	60.49%	/	TOTAL	381582788232	56.25%	/

Following the ranking of the out-degree and in-degree of agricultural products trade network along the route (Table 3), the out-degree of China, India, Indonesia, Russia, Thailand, and other countries have always been at the forefront of agricultural products trade network along the route in 2019 and 2021. Countries with certain advantages in agricultural products energy endowment or agricultural products energy utilization technology can export agricultural products to many countries in agricultural products trade along the route, which are relatively less affected by the COVID-19 epidemic. Thus, they wield relatively active exports in agricultural products trade network along the route.

Among them, China, Italy and Russia are all in the top three positions in the out-degree ranking of agricultural products trade network along the route, and the agricultural products trade activities are relatively active and are in a relatively central position in agricultural products trade. As far as in-degree is concerned, the in-degrees of China, Russia, India, Italy and other countries are also in the forefront in 2019 and 2021. When these countries export a large number of agricultural products, their demand for agricultural products is quite strong which allow them to occupy important position in the agricultural products trade network along the route. This may be due to their many kinds of agricultural products, along with the import and export of bulk agricultural products helping them to complement the demand and supply of agricultural products domestically and abroad. Notably, the out-degree and in-degree of agricultural products along the B&R rank among the top 10 countries such as China, Russia, Italy, and others. These countries occupy the most important positions in the agricultural product trade network along the B&R, indicating the most active countries in the agricultural products import and export trade, and have important influences at the same time. The active agricultural products trade with China and Russia may be due to their natural endowment factors in agricultural production, while the source of Italy's status as a big agricultural country is more owing to its high levels of domestic mechanization and its extensive popularization of new technologies. These allow Italy to stabilize its position in the agricultural products trade network along the route despite the impacts of COVID-19 on the global economy and agriculture.

#### 3.1.3. Modularity analysis

**Figure 4:**
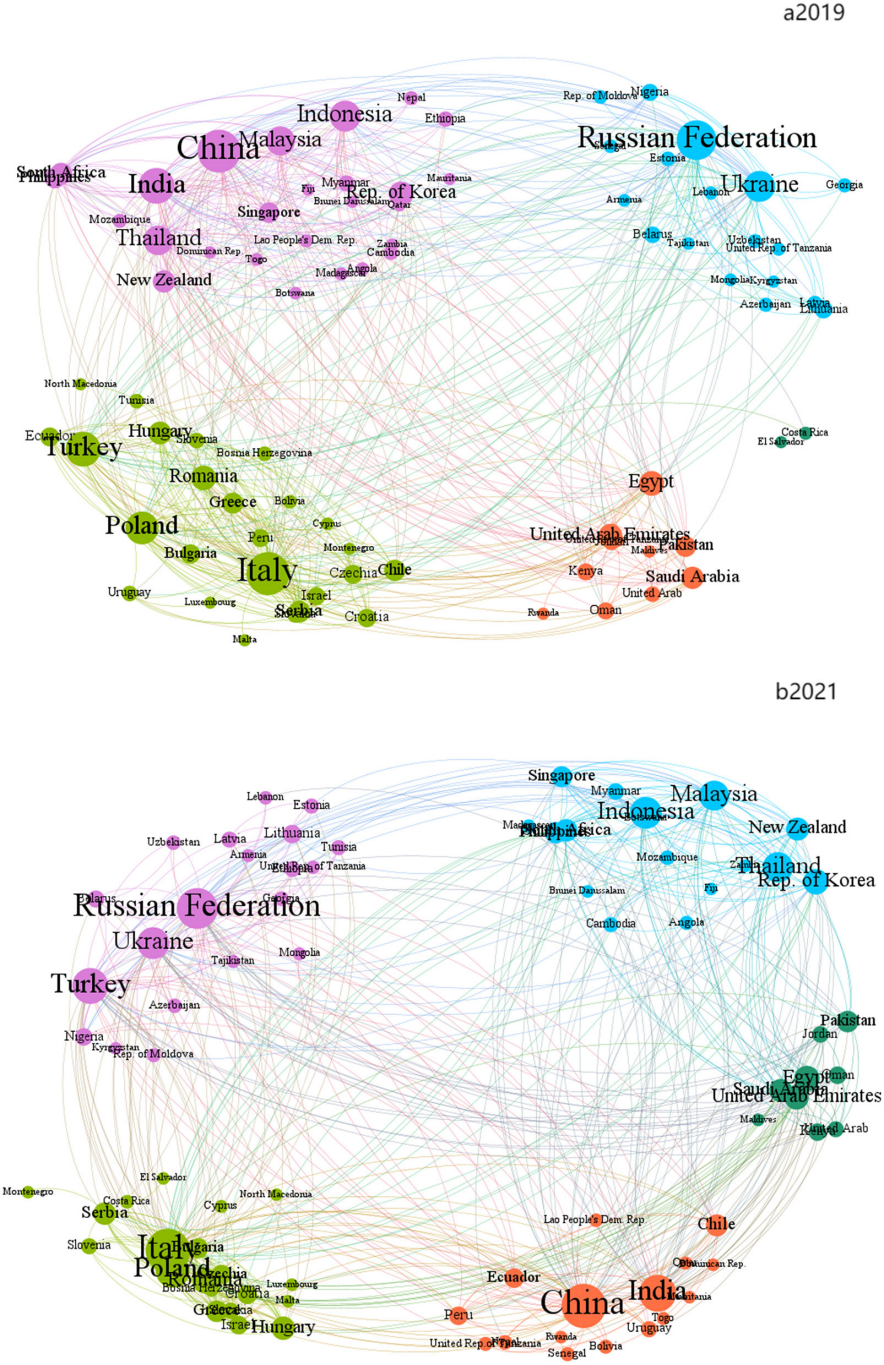
Community structure and flow patterns in the agricultural product. The same color indicates the same trade community. The thicker and larger the line and dot, the more scale of export quantity.

Modularity analysis of complex network is dividing the whole network into several sub-networks, with each sub-network being a community. Compared with the external connection, the internal association is closer. Modularity is an important index which measures the division of network associations—and there are many methods to divide the modules. Louvain algorithm was used to divide the modules of B&R agricultural products trade network in 2019 and 2021, respectively to observe the impact of COVID-19 epidemic on the modular evolution of the network. This included the edge weight in the calculation steps, making it more realistic. Comparing the community division map of agricultural products trade in countries along the B&R in 2019 and 2021, the internal community structure of agricultural products trade network along the route were found to experience the process of differentiation, reorganization, and re-differentiation before and after the outbreak of COVID-19. These mainly manifested in the following aspects:

From the perspective of the number of community members, the network in the two periods is all divided into five modules (Figure 4). Nevertheless, the number of core members led by the community has changed significantly. The number of node countries of the three major communities with China and the United Arab Emirates as the core has dropped significantly, and the distribution of members within the community is looser, showing a trend of a small group from a concentrated large community to a uniform one. The change of this situation may be due to the COVID-19 pandemic, the economic blockade policies of various countries restrict the trade of agricultural products across geographical distances and regions in most countries. Hence, they have to be constrained by geographical restrictions and therefore choose the nearest import and export markets. Simultaneously, with the unstable international situation, countries are more likely to choose relatively stable regional cooperation groups for trade to reduce the uncertain risks of cross-regional trade.

Judging from the attributes of the members of the internal community, the internal members of the five major communities have distinct geographical distribution characteristics, such as Asian community represented by China and India, the Eastern European community dominated by Russia and Ukraine, and the European community dominated by Italy and Poland (Figure 4). The members of these communities are all geographically close and have similar cultural and regional characteristics. Even during the pandemic, the composition factors of the communities linked by geopolitical and cultural characteristics have not significantly changed.

From the perspective of the evolution of community members, members Costa Rica and El Salvador in 2019 merged into the larger European community in 2021, forming a South Asian community with Malaysia at the core (Figure 4). South Asia is rich in tropical agricultural resources and banks on its natural factors, especially palm oil, rubber, tropical fruits, and other related products. Before the pandemic, agricultural products import and export markets in South Asia were dominated by the larger Asian market, making it part of the Asian modules in the early stage with China as its core. Nonetheless, to lessen the detrimental effects of epidemic control on the economy, countries in South Asia started focusing their trade on adjacent markets with comparatively lax epidemic control, which may have been inspired by China's severe COVID-19 epidemic control policies.

From the perspective of the degree of dependence within communities (Table 4), in 2019, the community with China as the core had the most absolute dependence, with China having the most absolute dependence (26). Among other communities, there were at least 7–8 pairs of absolute dependence in other communities, except the community composed of Costa Rica and El Salvador. But in 2021, the absolute dependence of community with China as the core decreased to 16. Even among the largest communities with Russia as the core, Russia's absolute dependence only reached 21 node countries, which was related to the formation of community with South Asia as the core. Countries with Malaysia as the core in South Asia also gradually separated from those with China to form new community.

**Table 4:** Characteristics of the agricultural products trade network community structure.

**2019**			**2021**		
**Community**	**Proportion (%)**	**Node**	**Community**	**Proportion (%)**	**Node**
China-core	30.95%	26	RUS-core	24.39%	21
Italy-Core	29.76%	25	Italy-core	24.39%	21
RUS-core	23.81%	20	Malaysia-core	20.73%	14
UAE-core	13.1%	11	China-core	19.51%	16
Coata Rita-core	2.38%	2	UAE-core	10.98	11

Generally, there is a direct relationship between the external dependence among countries and the import concentration of agricultural products: strong dependence leads to high import concentration, and the external supply of agricultural product in importing countries is more susceptible to COVID-19 risk in a single country. Meanwhile, when core exporting nations are more vulnerable to pandemics, it is more likely that other dependent nations' external agricultural product supply risk will rise. This is especially true if more countries within a community develop absolute dependence on a single nation, which also explains the change of community structure after the onset of COVID-19. Due to the pandemic, countries along the B&R increased their awareness of avoiding external uncertain risks, changed their agricultural trade strategies, and gradually reduced economic losses caused by the uncertain external supply risks represented by pandemic and its aftershocks.

### 3.2. Risk simulation and analysis

#### 3.2.1. Spatial pattern of risk factors

**Figure 5:**
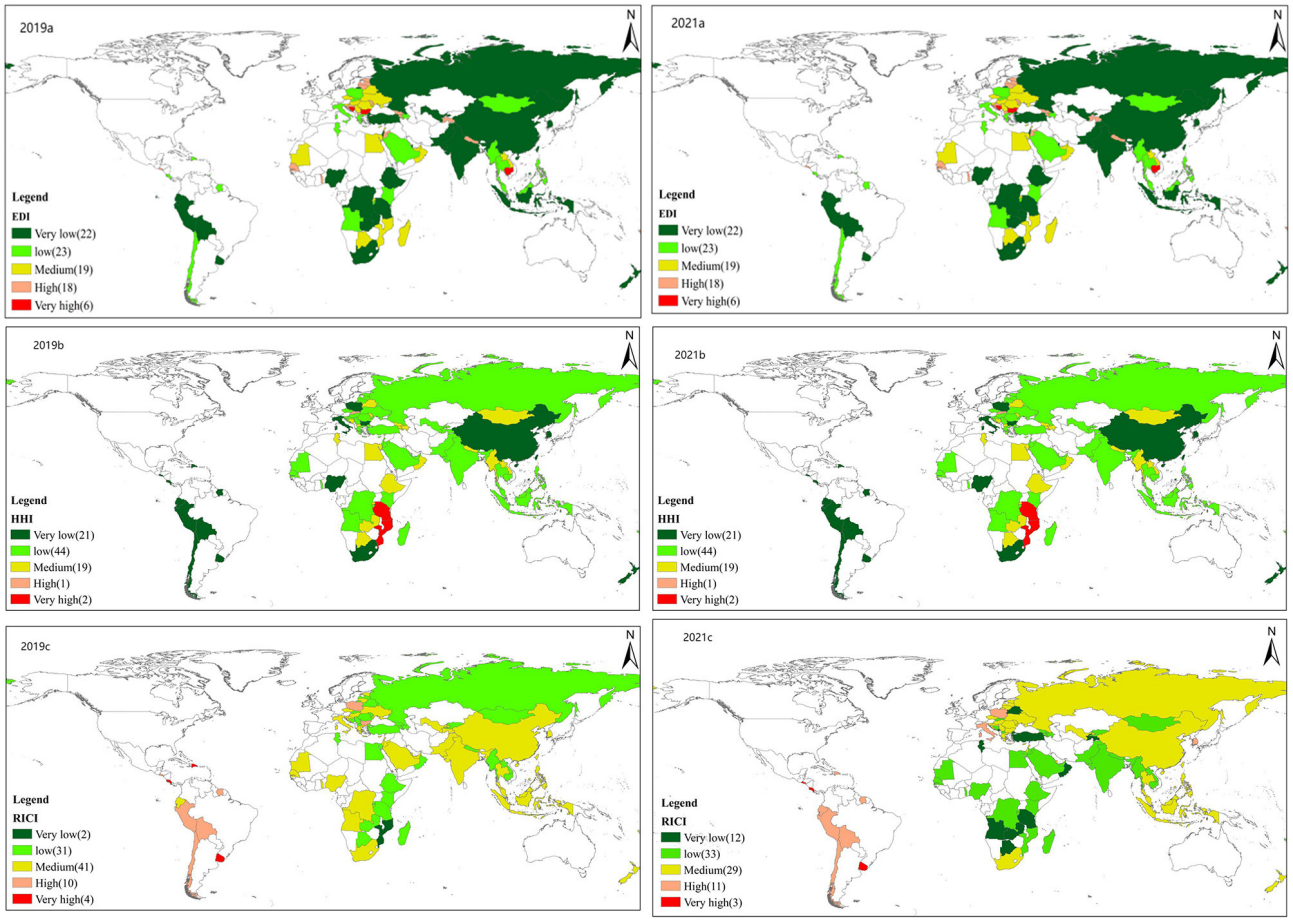
Spatial pattern of Agricultural products security risks. a, EDI; b, HHI; c, RICI in 2019 and 2021.

According to the formulas of EDI, HHI and RICI above, this study calculates three major risk indicators: (1) The EDI results show that in 2019, more than 70% of all countries (64 countries) along the B&R were at either moderate or below risk (Figure 5 2019a), 24 were at high-risk or above, and 18 and 6 countries with high-risk and extremely high-risk, respectively and were mainly distributed in Europe and South Asia. In 2021, along the B&R 59 countries had medium and low risks while 25 had high and extremely high risks (Figure 5 2021a) and were mainly distributed in Europe and Africa; (2) HHI results show that in 2019 and 2021, 85 countries were at low to medium-risk level (Figure 5, 2019b and 2021b). Nevertheless, compared with 2019, in 2021, the number of countries with extremely low-risk decreased by 4 and the number of countries with medium-risk increased by 6, with most of them distributed in southern and northern Africa while the number of those distributed in other grades being relatively stable (Figure 5 2021b). (3) RICI results show that countries with extremely low-risk level decreased from 12 in 2019 to 2 in 2021, while countries with medium-risk level increased from 29 to 41, and were mainly located in South Asia, Africa, and Latin America (Figure 5 2019c, 2021c).

Notably, Russia changed from a medium-risk level country to a low-risk one. This may be due to two reasons: first, from the perspective of import supply risk, Russia is a big exporter of agricultural products: as the main supplier of agricultural products export, it has a strong bargaining power in the global market. Russia has therefore gained a market advantage especially during the onset of the pandemic where food security was an important issue. Second, many countries with relatively small economies along the B&R are excessively dependent on the import of agricultural products and their import trade deficit of agricultural products have perennially existed, making them more susceptible to the export control measures of agricultural products caused by the pandemic. Hence, countries with relatively small economies stayed in the relatively high-risk range in the ArcGIS natural discontinuity method, compared to Russian which changed from medium-risk to low-risk. In general, comparing the number of countries with different risk levels of various risk indicators before and after the onset of COVID-19, the risk levels of most countries along the route have improved after the pandemic. Nonetheless, its negative effects cannot be underestimated.

#### 3.2.2. Spatial pattern of comprehensive risk

**Figure 6:**
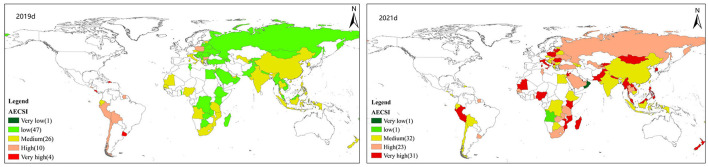
Spatial pattern of Comprehensive Risk of agricultural products security risks. d, AECSI in 2019 and 2021.

Because of the pandemic, AESCI in countries along the B&R has changed to varying degrees, with the AECSI in most countries significantly increasing (Figure 6 2019d, 2021d). Overall, the average value of AECSI increased from 4.711 in 2019 to 117.23 in 2021, indicate a more than 100% increase. Evidently, it is vitally important to analyze the impact of COVID-19 by including the COVID-19 risk index in the external risk supply model. From a community perspective, the average AECSI value of Italy-Core community is the highest, which is higher than the average level of B&R. This is followed by the Russia-Core community and the UAE-Core community. At the national level, compared to 2019, the number of countries with low-risk level dropped from 47 to 1, the number of countries with medium-risk level rose from 26 to 32, the number of countries with high-risk level rose from 10 to 23, and the number of countries with extremely high-risk level rose to 31. Among them, countries with high-risk level and extremely high-risk level are mainly distributed in Africa, Europe, and some Pacific Island countries, while countries with medium-risk level are mainly distributed in eastern Asia, south Asia and central Africa.

Among the top 10 import countries in 2021, 5 countries (Russia, United Arab Emirates, Turkey, South Korea, Italy) are at high or extremely high-risk level, while the remaining 5 countries are at medium-risk level. Generally, due to the global effects of COVID-19, the stability of the original international market of agricultural products has been broken, the external supply risk of agricultural products became increasingly worrying, and food security was given greater socioeconomic and political attention.

#### 3.2.3. The identification of the dominant risk

**Figure 7:**
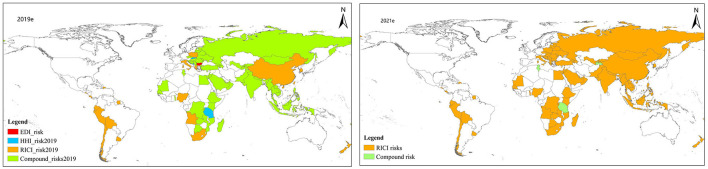
Spatial pattern of the dominant risk of Agricultural products security in 2019 and 2021.

After identifying the dominant risk types in 2019 and 2021, it is found that in 2019, countries with EDI, HHI, RICI and compound risk were all distributed, especially countries with compound risk types accounting for more than 80% being mostly distributed in Europe, South Asia, Oceania, and other continents (Figure 7 2019e, 2021e). AECSI in these countries were mostly at low-risk and medium-risk level. This meant that a single risk may lead to more serious external supply risks. Two countries belonged to HHI risk (Tanzania and Slovenia), while Bulgaria belonged to EDI risk type. Following the COVID-19 outbreak, RICI became the main danger along the B&R, composed of three nations' remaining compound hazards. These nations' initial AECSI risk score for 2019 typically varied between 3.0 and 4.5, while the risk index after the COVID-19 outbreak soared to 50–100.

The pandemic's outbreak arguably changed the external supply risk of agricultural products along the route, especially changing the original compound-dominated compound risk type along with the epidemic risks caused by the epidemic impacted the countries along the route, which is largely consistent with the distribution number of countries with medium and high risks in AECSI. Clearly, the impact of COVID-19 epidemic on agricultural products trade in B&R and the impact on external supply of agricultural products can be observed by incorporating COVID-19 risk index into the external supply risk model and combining the agricultural products trade data before and after the epidemic, making a data test for countries along the route to change their trade strategies.

## 4. Discussion

Following the complex network perspective, this study uses the agricultural products import trade data of 88 countries along the B&R from the UN comtrade database in 2019 and 2021. Based on the complex network method, it identifies the spatial correlation structure, core nodes, and module distribution of agricultural products trade network in B&R under the COVID-19 global situation, and analyzes the evolution of various characteristics of agricultural products trade such as the flow pattern and interdependence among and within sections. It also constructs a risk assessment model (comprised of EDI, HHI, and RICI) of external supply of agricultural products in countries along the route, and analyzes the impact of COVID-19 epidemic on the external supply risk changes *in toto*, as well as the changes of the dominant risk types before and after the pandemic.

Analyzing the agricultural products trade network structure of countries along the B&R, the following results are shown: first, ever since the outbreak of the pandemic in 2020, the trade spatial correlation structure of B&R has gradually loosened and the overall contact density has also decreased. This change is further reflected in the modularity analysis, where the agricultural products export along the route has begun to turn to countries with relatively loose epidemic control and relatively close geographical location, thus the trend of grouping based on geopolitics reappears.

Second, from the calculation results of degree distribution, the random probability distributions of degree, out-degree, and in-degree all show a downward trend, and the scale-free distribution characteristics are clear. A few core node countries also play the role of pacemakers. However, under the multiple influences of the pandemic along with other external conditions, the loss of core node countries' status easily affect the stability of the entire network and accelerates its collapse (36), which must also be a key consideration in the evaluation of external supply risks herein. The closer the connection, the higher the vulnerability of the network layout of a few node countries under the impact of unexpected external risks.

Third, results of external supply risk assessment, based on the comparison of EDI, HHI, RICI and RECSI in 2019 and 2021 reveals that the pandemic has significantly affected the import dependence, import concentration, and risk level of COVID-19 in countries along the B&R, with the pandemic becoming the main risk source affecting the supply of agricultural products along the route.

## 5. Limitations

The impact of COVID-19 epidemic on the global supply of agricultural products and food security is multifaceted. According to data from the World Bank, the prices of most agricultural products affected by the epidemic continued to rise, especially after the outbreak of the conflict between Russia and Ukraine, global agricultural prices soared further. At the same time, with the global population breaking through the 8 billion mark and the rapid progress of urbanization, the problem of agricultural product supply chain is becoming more and more complicated. In this article, only the COVID-19 epidemic index and trade-related index were taken into account in setting the external supply risk model, while the evaluation perspective can be further extended. Future research can more comprehensively consider the impact of COVID-19 and other factors on the external supply of global agricultural products and global food security, and then put forward effective countermeasures.

## 6. Conclusion

The external supply risk of agricultural products exporting countries along the route mainly comes from the epidemic control of importing countries. To maintain the stability of agricultural products trade network along the B&R and reduce the external supply risk, it is necessary to maintain the liquidity of agricultural products along the B&R and avoid excessively restrictive trade measures, especially for core countries which are very important for food security along the route. Timely adjustment of strategy and improvement of domestic supply level will help to reduce external dependence risk (R_EDI_), while getting rid of community restrictions and developing emerging import markets will help to reduce import concentration risk (R_HHI_) and COVID-19 epidemic risk (R_RICI_). Strengthening food aid is an important measure to deal with the severe global COVID-19 situation, and in the long run, the application of digital technology will play a huge role in increasing domestic agricultural capacity and ensuring domestic agricultural supplies.

## Data availability statement

The data presented in the study are deposited in the UN comtrade repository, published data of Green Belt and Road Initiative Center (https://greenfdc.org/green-belt-and-road-initiative-bri-lab/); covid19-risk-index.com. The original contributions presented in the study are included in the article/Supplementary material, any further enquiries or requests to access undisclosed data should be directed to the corresponding author.

## Author contributions

Conceptualization, methodology, and draft writing: QH, MG, and FW. Validation: QH and LS. Review and editing: QH, LS, and XW. Check of layout: XW. All authors have read and agreed to the published version of the manuscript.

## Footnotes

^1^Fourty sub-Saharan African countries, 34 European and Central Asian countries (including 18 EU countries), 25 East Asian and Pacific countries, 17 Central Asian and North African countries, 18 Latin American and Caribbean countries and 6 Southeast Asian countries.

^2^Angola, Armenia, Azerbaijan, Barbados, Belarus, Bolivia, Bosnia and Herzegovina, Brunei, Bulgaria, Burund, Chile, Congo, China, Dem. Rep, Costa Rica, Croatia, Cyprus, Czech Republic, Ecuador, Egypt, El Salvador, Estonia, Ethiopia, FIJI, Gambia, Georgia, Ghana, Greece, Grenada, Hungary, Indonesia, Italy, Kenya, Korea, Kyrgyzstan, Laos, Latvia, Lebanon, Lithuania, Luxembourg, Madagascar, Malaysia, Maldives, MALTA, Mauritania, Moldova, Mongolia, Montenegro, Mozambique, Myanmar, Nepal, New Zealand Nigeria, Macedonia, Oman, Pakistan, Peru, Philippines, Poland, Qatar, Romania, Russia, Rwanda, Samoa, Saudi Arabia, Senegal, Serbia, Singapore, Slovakia, Slovenia, South Africa, Sri Lanka, Suriname, Tajikistan, Tanzania, Thailand, Togo, Trinidad and Tobago, Tunisia, Turkey, Ukraine, United Arab Emirates, Uruguay, Uzbekistan, Zambia.

## References

[1] Huang H Lanpe M V Tongereen F V. Climate change and trade in agriculture. Food Policy. (2011) 36:S9–S13. 10.1016/j.foodpol.2010.10.008

[2] Zhang C Yang YZ Feng ZM Xiao CW Lang TT Du WP. Risk of global external cereals supply under the background of Covid-19 Pandemic: based on the perspective of Trade Network. Foods. (2021) 10:1168. 10.3390/foods10061168 34071044 PMC8246323

[3] Akbari M Foroudi P Shahmoradi M Padash H Parizi ZS Khosravani A. The evolution of food security: where are we now, where should we go next? Sustainability. (2022) 14:3634. 10.3390/su14063634

[4] Ataei P Sadighi H Izadi N. Major challenges to achieving food security in rural, Iran. Rural Soc. (2021) 30:15–31. 10.1080/10371656.2021.1895471

[5] Watts DJ Strogatz S H. Collective dynamics of small-world networks. Nature. (1998) 393:440–2. 10.1038/30918 9623998

[6] Newman M. The structure and function of complex networks. J Siam Rev. (2003) 45:167–256. 10.1137/S003614450342480

[7] Li B Wang H Xiao X. Measuring financial contagion using general social interaction model with trade network structure. Appl Econ Lett. (2014) 21:631–635. 10.1080/13504851.2013.879279

[8] Lee J Yun K Jin L. China's New Silk Road: policies and implications. J Int Logist Trade. (2015) 13:55–70. 10.24006/jilt.2015.13.2.55

[9] Chen S Qiang Q P. The trade network structure of the “one belt and one road” and its environmental effects. Sustainability. (2020) 12:1–21. 10.3390/su1209351935136666

[10] Liu LQ Chen ZR Tian BF. How does structural dependence affect the formation and evolution of trade network: taking “The Belt and Road initiative” as an example. World Econ Res. (2020) 137:106–120. 10.13516/j.cnki.wes.2020.06.009

[11] Li YY Li YL Pan A Pan X Veglianti E. The network structure characteristics and determinants of the belt & road industrial robot trade. Emer Markets Finance Trade. (2021) 58:1491–501. 10.1080/1540496X.2021.1897315

[12] Wang X Ge J Wei WD Li HS Wu C Zhu G. Spatial dynamics of the communities and the role of major countries in the international rare earths trade: a complex network analysis. PLoS ONE. (2016) 11:e0154575. 10.1371/journal.pone.0154575 27137779 PMC4854386

[13] Wu AP Zhang XP Song XF Li RK. Global nuclear power equipment trade network structure and its influencing factors from 2000 to 2019. Econ Geogr. (2022) 42:126–134. 10.15957/j.cnki.jjdl.2022.07.013

[14] Wang F Hu Q G. Analysis of the structure of the trade network of mechanical and electrical products in countries along the B&R. Asia-Pacific Econ. (2019) 150:49–58. 10.16407/j.cnki.1000-6052.20191025.005

[15] Fan Y Ren S Cai H Cui XF. The State's role and position in international trade: a complex network perspective. Econ Model. (2014) 39:71–81. 10.1016/j.econmod.2014.02.027

[16] Ma S Z Ren W Wu G J. The Characteristics of a country's agricultural trade network and its influence on the division of labor in the global value chain— based on the perspective of social network analysis. Manage World. (2016) 3:60–72. 10.19744/j.cnki.11-1235/f.2016.03.006

[17] Nie C L Jiang H N Duan J. The spatial pattern evolution of global grain trade network since the 21st century. Econ Geogr. (2021) 41:119–27. 10.15957/j.cnki.jjdl.2021.07.013

[18] Wu F Guclu H. Global maize trade and food security: implications from a social network model. Risk Analy. (2013) 33:2168–78. 10.1111/risa.12064 23656551 PMC3762915

[19] Sartori M Schiavo S. Connected we stand: a network perspective on trade and global food security. Food Policy. (2015) 11:114–27. 10.1016/j.foodpol.2015.10.004

[20] Zhou MZ Wang J. Evolution of global rice trade pattern based on complex network and its enlightenment. J Nat Resour. (2020) 35:13. Available online at: https://kns.cnki.net/kcms/detail/11.1912.N.20200525.1202.008

[21] Puma M J Bose S Chon S Y Cook B I. Assessing the evolving fragility of the global food system. Environ Res Lett. (2015) 10:024007. 10.1088/1748-9326/10/2/024007

[22] Hubbard L J Hubbard C. Food security in the United Kingdom: External supply risks. Food Policy. (2013) 43:142–7. 10.1016/j.foodpol.2013.08.006

[23] Gephart J A Rovenskaya E Dieckmann U Pace M L Brännström Å. Vulnerability to shocks in the global seafood trade network. Environ Res Lett. (2016) 11:035008. 10.1088/1748-9326/11/3/03500834513582

[24] Allevi E Boffino L De Giuli M E Oggioni G. Evaluating the impacts of the external supply risk in a natural gas supply chain: the case of the Italian market. J Global Optimiz. (2018) 70:347–84. 10.1007/s10898-017-0584-z

[25] Iqbal W Fatima A Hou Y Abbas Q Iram R. Oil supply risk and affecting parameters associated with oil supplementation and disruption. J Clean Prod. (2020) 255:120187. 10.1016/j.jclepro.2020.120187

[26] Coq C L Paltseva E. Measuring the security of external energy supply in the European Union. Energy Policy. (2009) 37:4474–81. 10.1016/j.enpol.2009.05.069

[27] Cao LJ Li TX Wang RB Zhu J. Impact of COVID-19 on China's agricultural trade. China Agric Econ Rev. (2021) 13:1–21. 10.1108/CAER-05-2020-0079

[28] Gleditsch KS. Expanded trade and GDP data. J Confl Resol. (2002) 46:712–24. 10.1177/002200202236171

[29] Zhan MH. Competitiveness and complementarity of agricultural products trade in countries along the B&R-based on social network analysis method. Agric Econ Issues. (2018) 2:103–14. 10.13246/j.cnki.iae.2018.02.012

[30] Shirley MD Rushton SP. The impacts of network topology on disease spread. Ecol Complex. (2005) 2:287–99. 10.1016/j.ecocom.2005.04.005

[31] Zhong ZH Qin CL. B&R trade network structure and its influencing factors-a study based on network analysis method. Int Econ Trade Explor. (2017) 33:15–28. 10.13687/j.cnki.gjjmts.2017.05.002

[32] Newman M E J Girvan M. Finding and evaluating community structure in networks. Phys Rev E. (2004) 69:026113. 10.1103/PhysRevE.69.026113 14995526

[33] Yang Y Li J P Sun X Chen J. Measuring external oil supply risk: a modified diversification index with country risk and potential oil exports. Energy. (2014) 68:930–8. 10.1016/j.energy.2014.02.091

[34] Wang X Qiang W L Niu S W Cheng S K Li Z. Global agricultural trade network and its evolution analysis. J Nat Resour. (2018) 33:940–53. Available online at: https://kns.cnki.net/kcms/detail/detail.aspx?FileName=ZRZX201806003&DbName=CJFQ2018

[35] Ji Q Zhang H Y Fan Y. Identification of global oil trade patterns: An empirical research based on complex network theory. Energy Conver Manage. (2014) 85:856–65. 10.1016/j.enconman.2013.12.072

[36] Crucitti P Latora V Marchiori M Rapisarda A. Error and attack tolerance of complex networks. Phys Statist Mech Applic. (2004) 1:388–94. 10.1016/j.physa.2004.04.03117930100

