# Supplementary figures and images for: External supply risk of agricultural products trade along the Belt and Road under the background of COVID-19
Source: Front Public Health. 2023 Feb 15;11:1122081. doi: 10.3389/fpubh.2023.1122081 (PMC9976227; doi:10.3389/fpubh.2023.1122081)

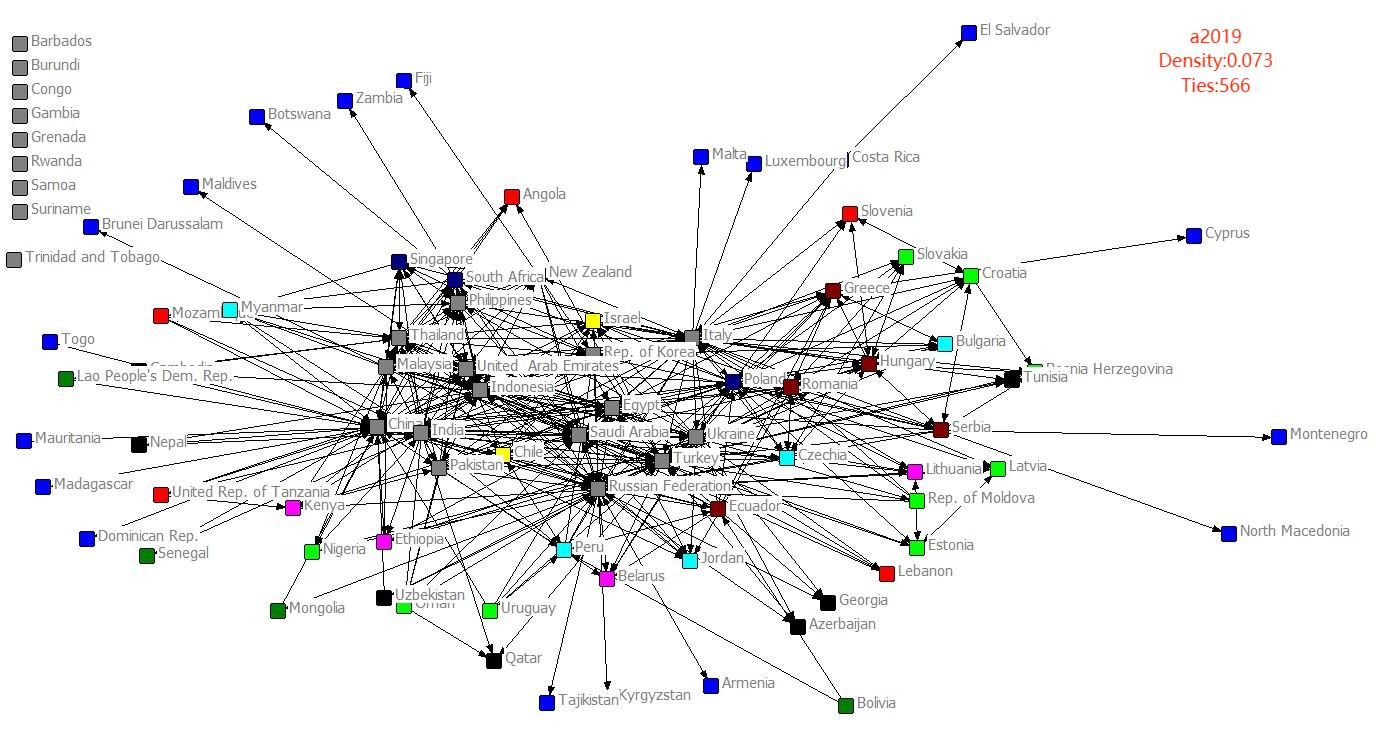

Supplement: Supplementary file 1 [file Data_Sheet_1.zip › DATA/Figure/Figure1-2019a.jpg]

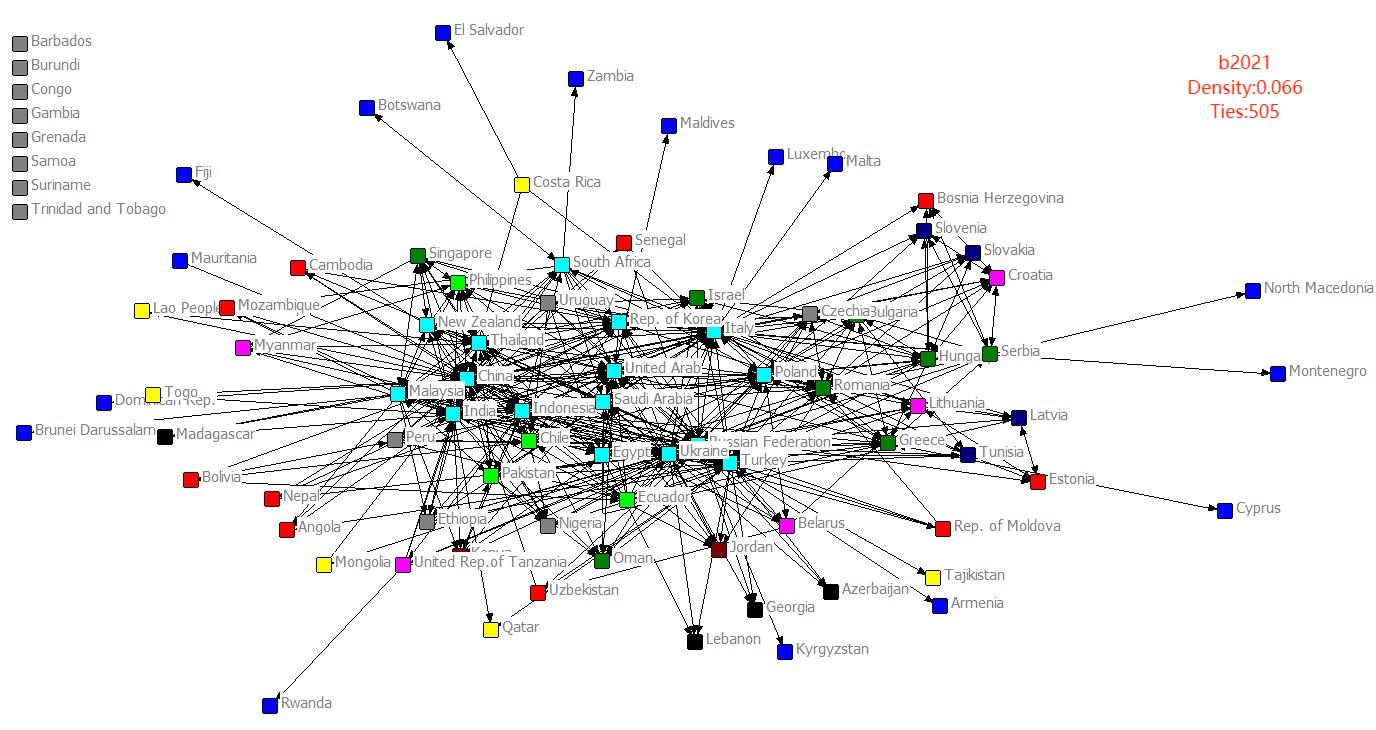

Supplement: Supplementary file 1 [file Data_Sheet_1.zip › DATA/Figure/Figure1-2021b.jpg]

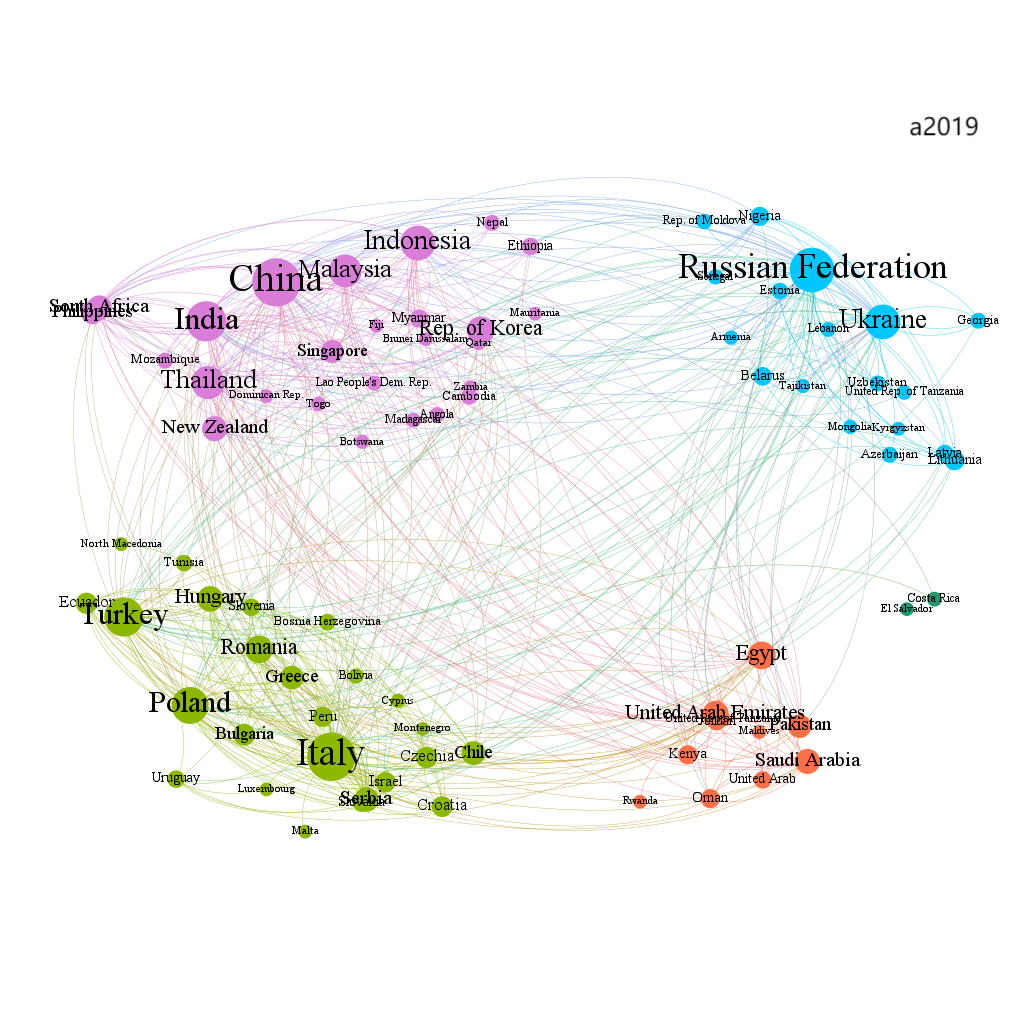

Supplement: Supplementary file 1 [file Data_Sheet_1.zip › DATA/Figure/Figure5-a2019.png]

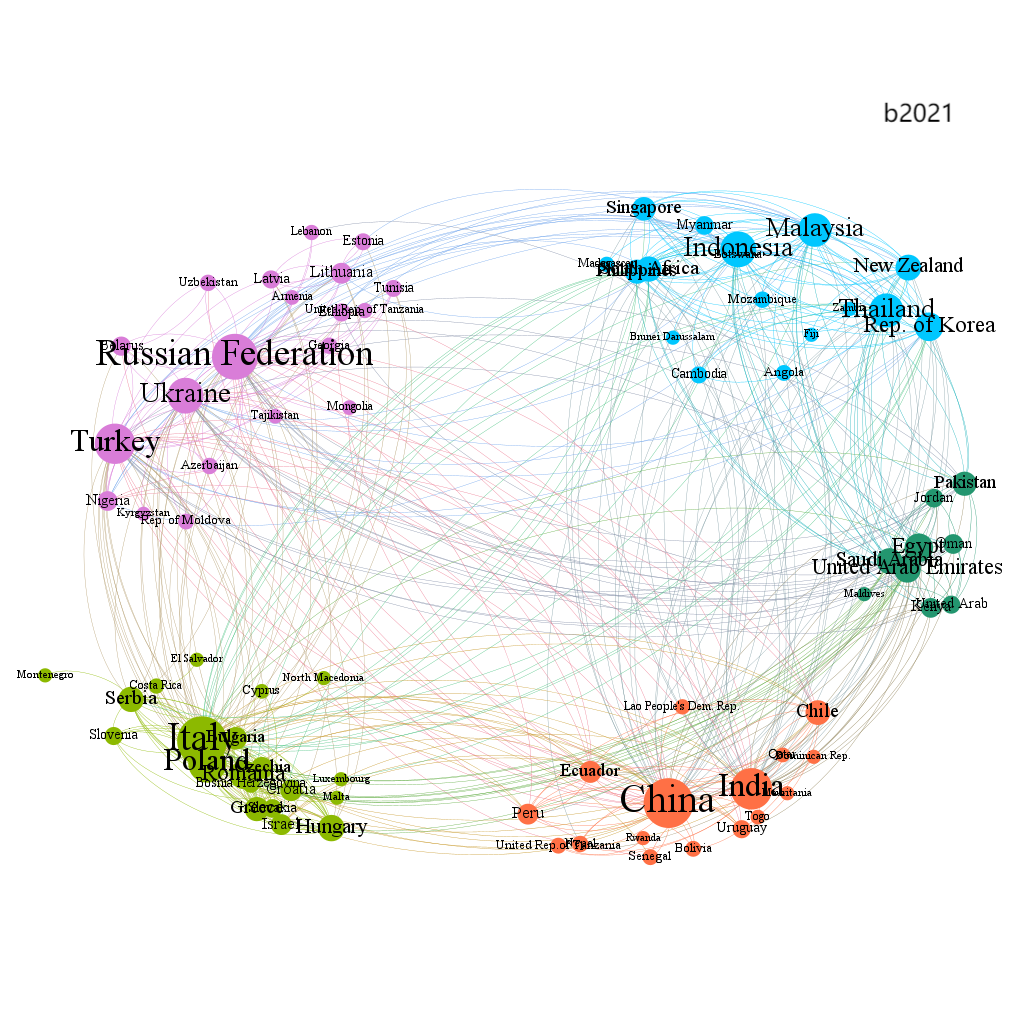

Supplement: Supplementary file 1 [file Data_Sheet_1.zip › DATA/Figure/Figure5-b2021.png]

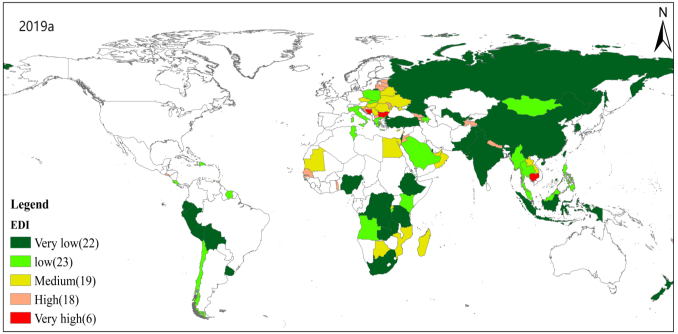

Supplement: Supplementary file 1 [file Data_Sheet_1.zip › DATA/Figure/Figure6-2019a(EDI).png]

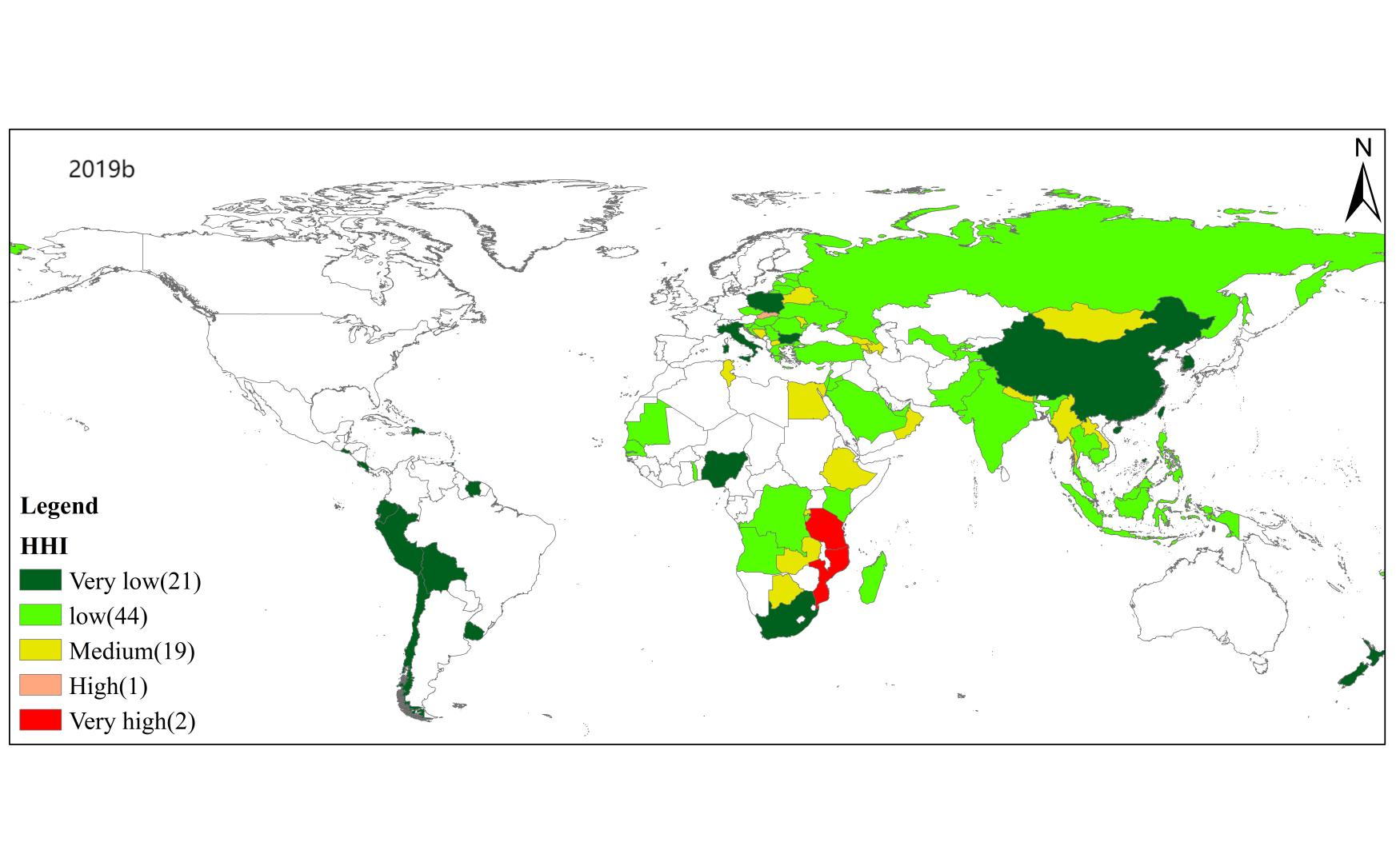

Supplement: Supplementary file 1 [file Data_Sheet_1.zip › DATA/Figure/Figure6-2019b(HHI).jpg]

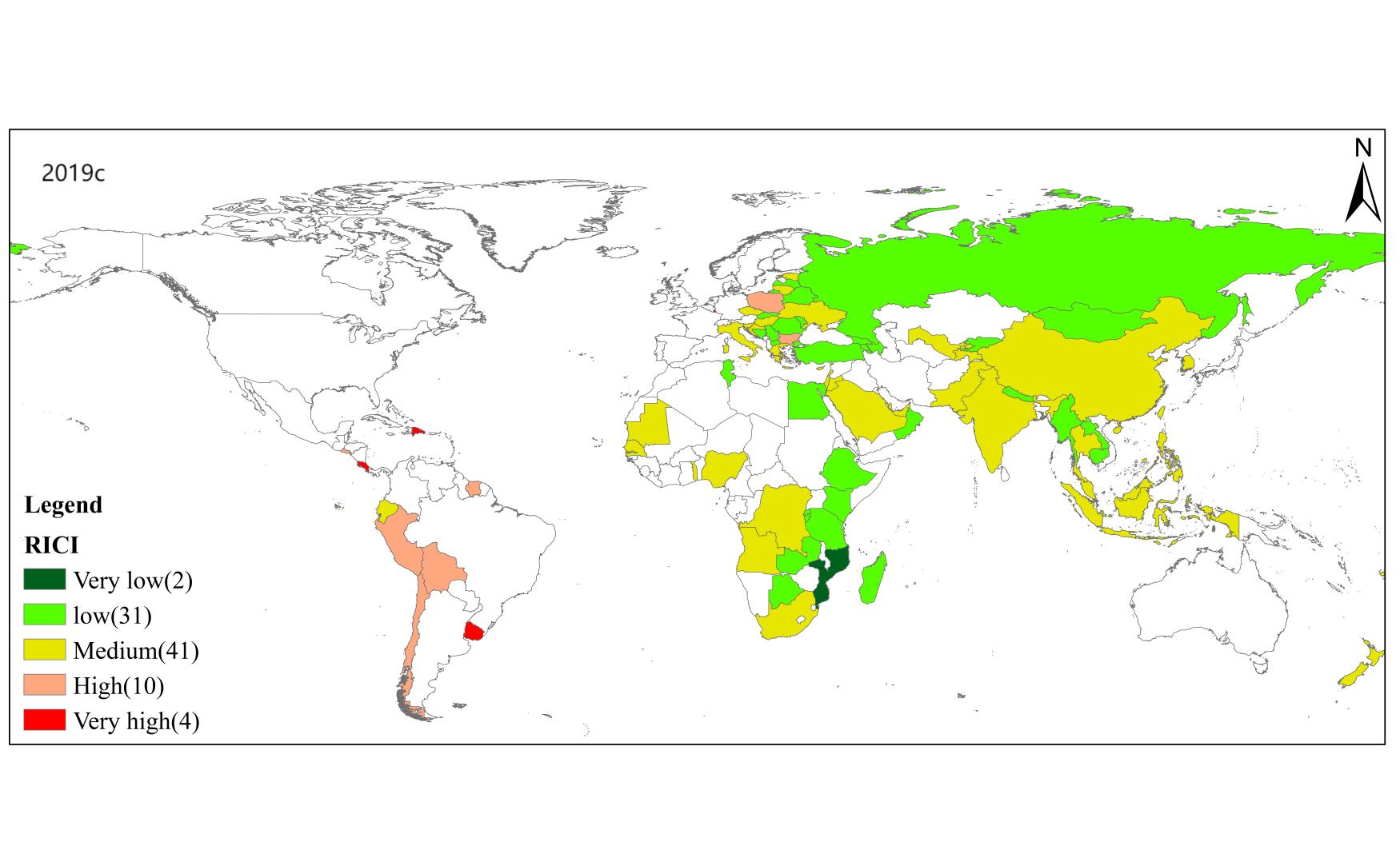

Supplement: Supplementary file 1 [file Data_Sheet_1.zip › DATA/Figure/Figure6-2019c(RICI).jpg]

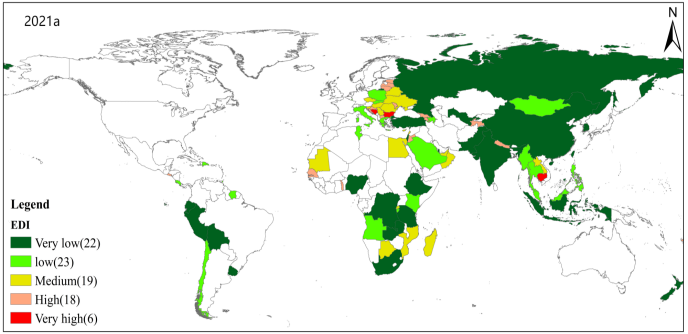

Supplement: Supplementary file 1 [file Data_Sheet_1.zip › DATA/Figure/Figure6-2021a(EDI).png]

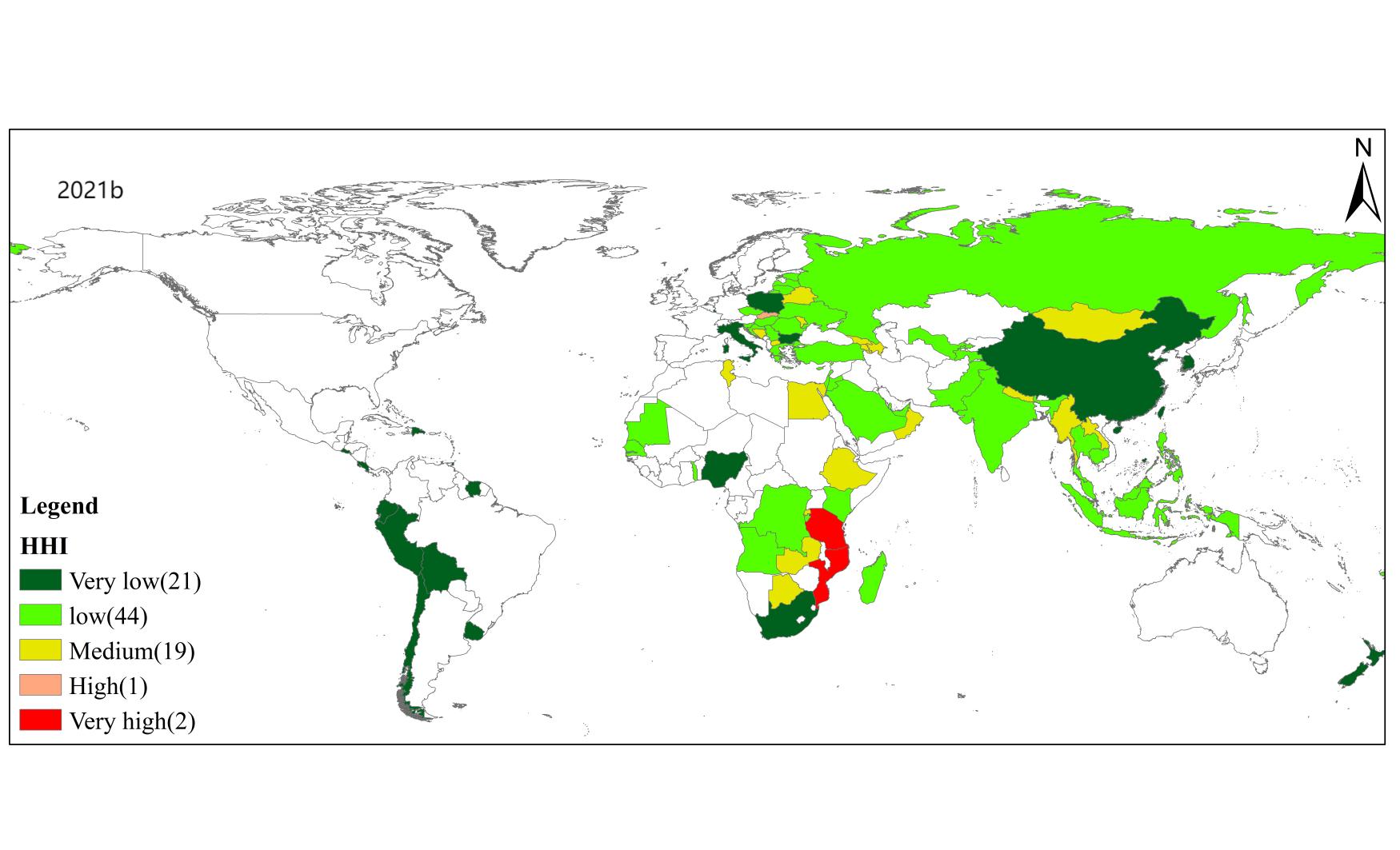

Supplement: Supplementary file 1 [file Data_Sheet_1.zip › DATA/Figure/Figure6-2021b(HHI).jpg]

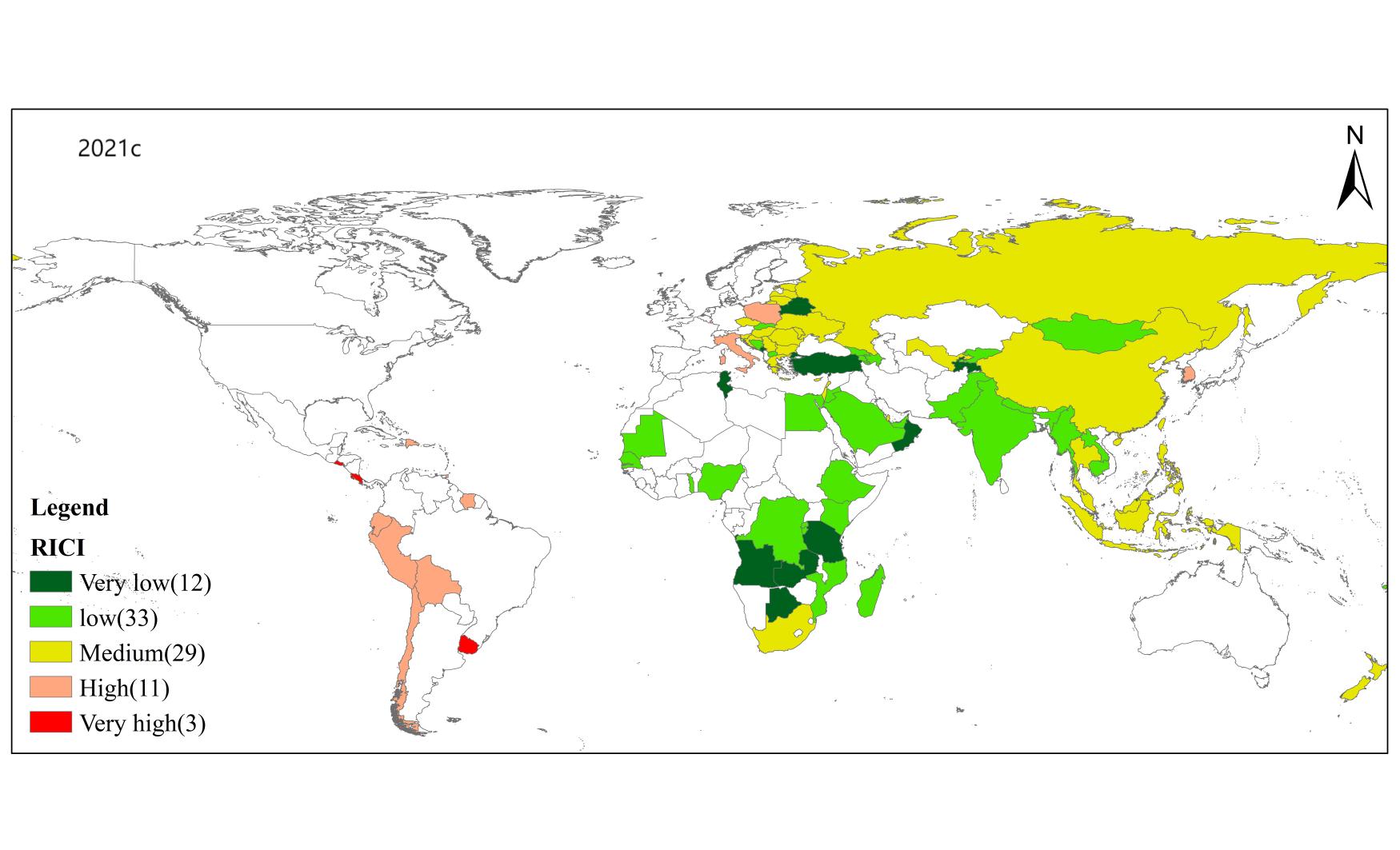

Supplement: Supplementary file 1 [file Data_Sheet_1.zip › DATA/Figure/Figure6-2021c(RICI).jpg]

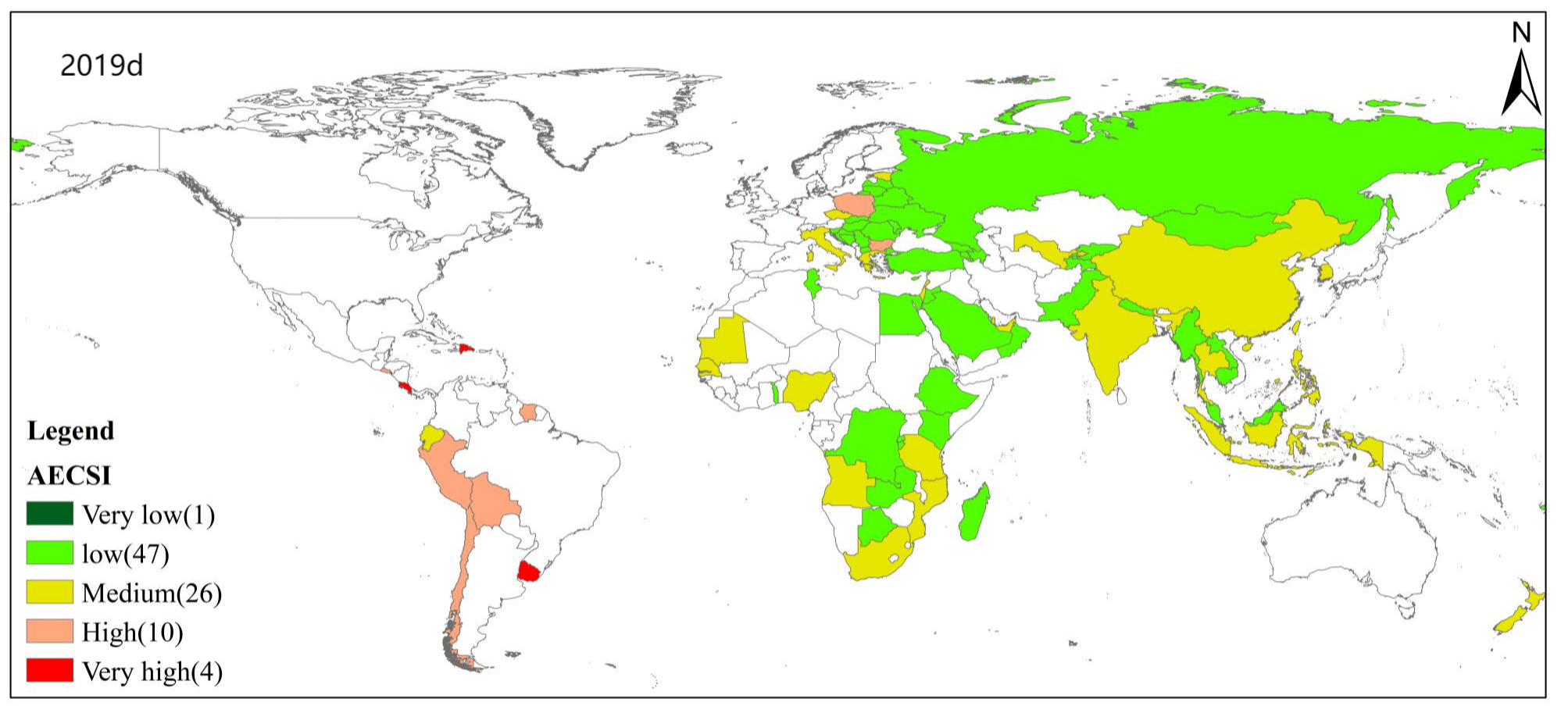

Supplement: Supplementary file 1 [file Data_Sheet_1.zip › DATA/Figure/Figure7-2019d(AECSI).png]

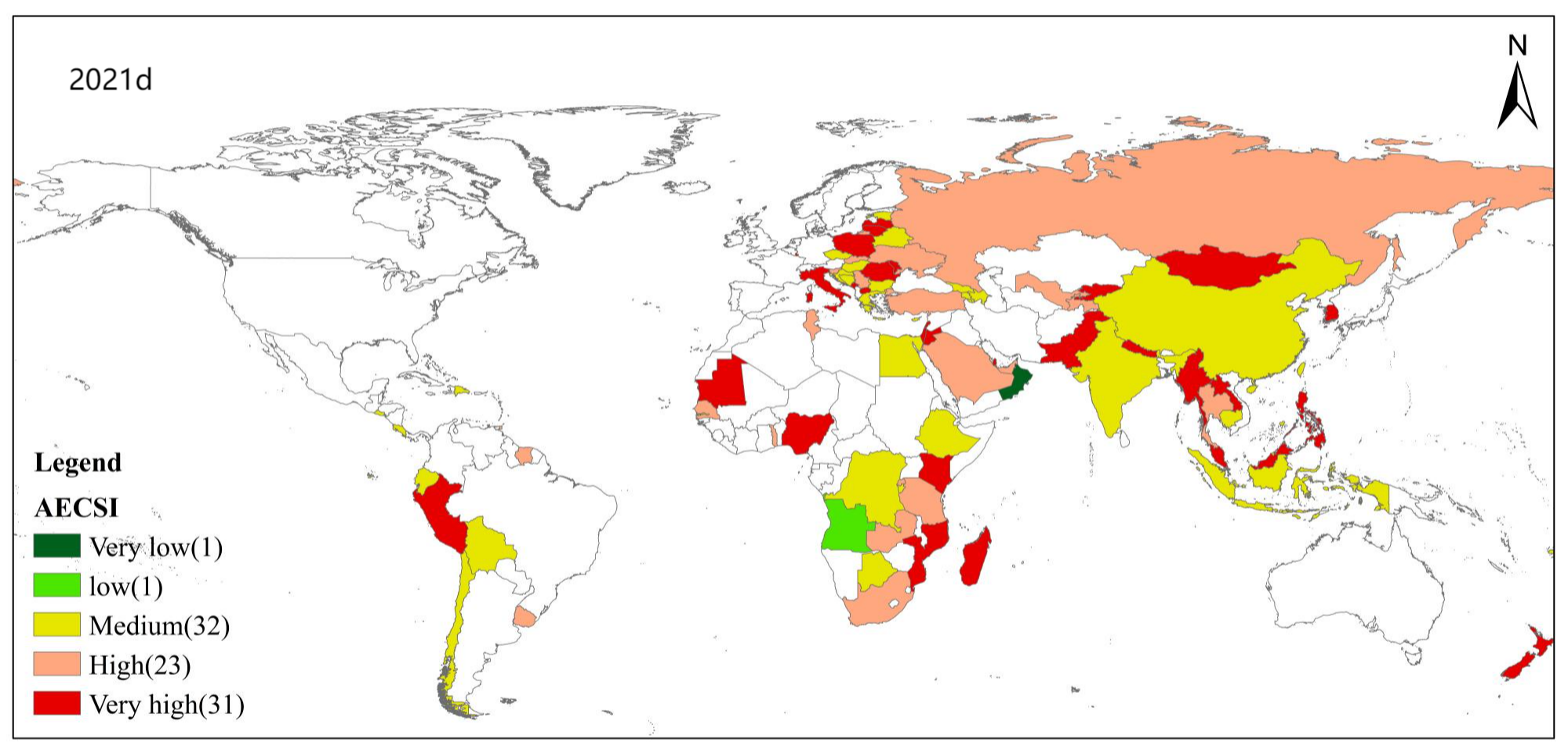

Supplement: Supplementary file 1 [file Data_Sheet_1.zip › DATA/Figure/Figure7-2021d(AECSI).png]

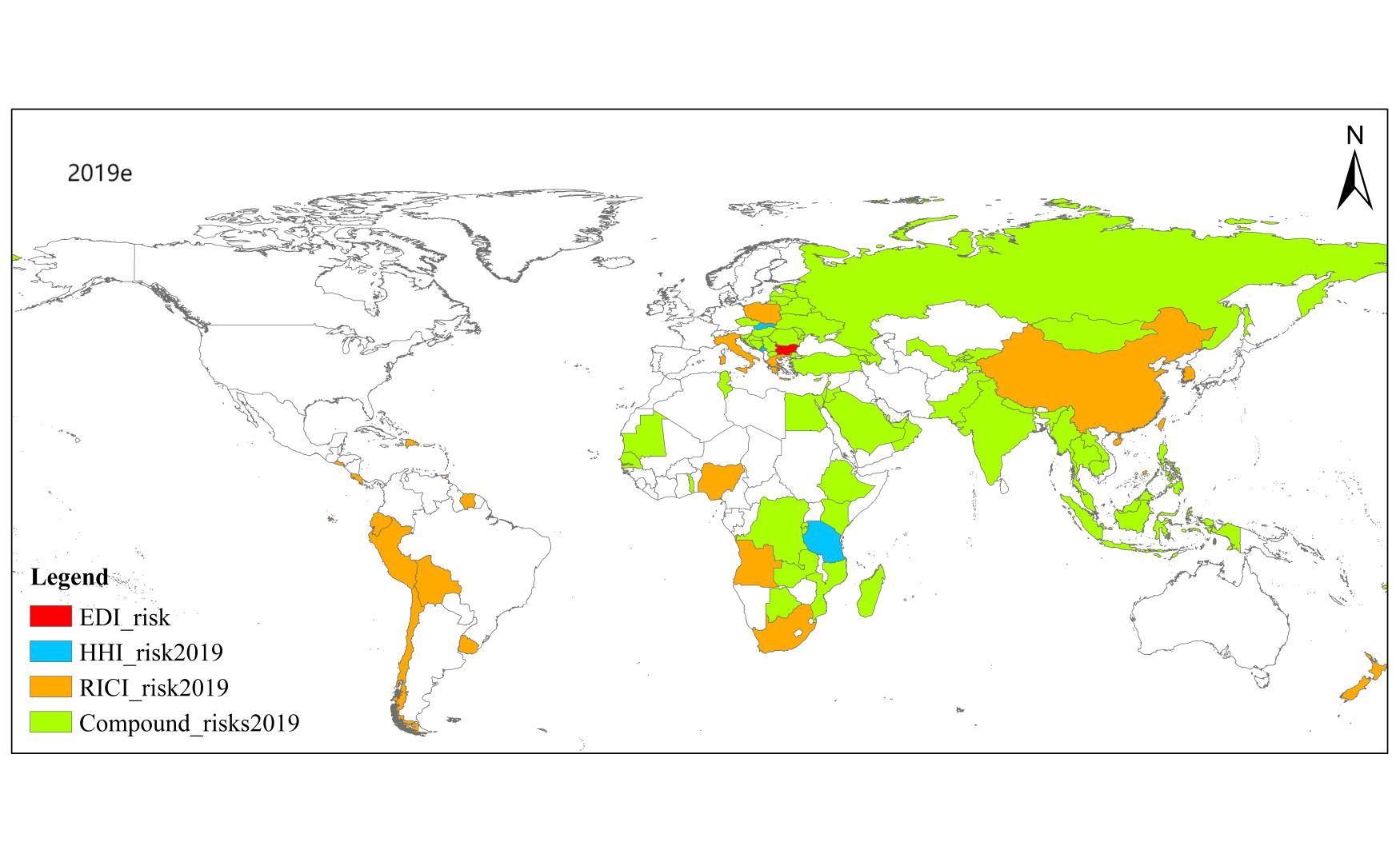

Supplement: Supplementary file 1 [file Data_Sheet_1.zip › DATA/Figure/Figure8-2019e(dominant risk.jpg]

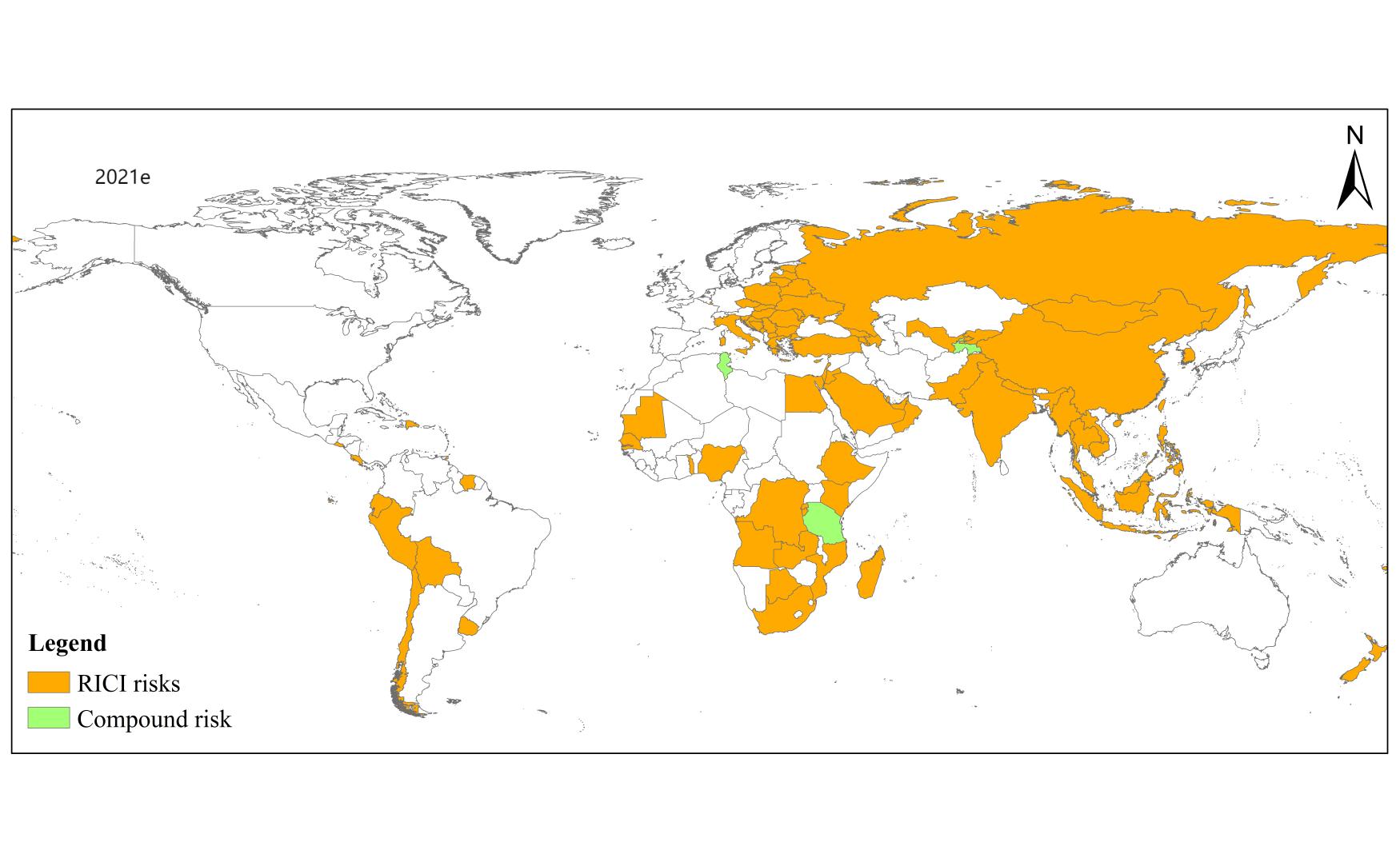

Supplement: Supplementary file 1 [file Data_Sheet_1.zip › DATA/Figure/Figure8-2021e(dominant risk).jpg]
